# New frontiers for the materials genome initiative

**DOI:** 10.1038/s41524-019-0173-4

**Published:** 2019

**Authors:** Juan J. de Pablo, Nicholas E. Jackson, Michael A. Webb, Long-Qing Chen, Joel E. Moore, Dane Morgan, Ryan Jacobs, Tresa Pollock, Darrell G. Schlom, Eric S. Toberer, James Analytis, Ismaila Dabo, Dean M. DeLongchamp, Gregory A. Fiete, Gregory M. Grason, Geoffroy Hautier, Yifei Mo, Krishna Rajan, Evan J. Reed, Efrain Rodriguez, Vladan Stevanovic, Jin Suntivich, Katsuyo Thornton, Ji-Cheng Zhao

**Affiliations:** 1 Institute for Molecular Engineering, University of Chicago, Chicago, IL 60615, USA; 2 Department of Materials Science and Engineering, The Pennsylvania State University, University Park, PA 16802, USA; 3 Department of Physics, University of California, Berkeley, Berkeley, CA 94720, USA; 4 Department of Materials Science and Engineering, University of Wisconsin-Madison, Madison, WI 53706, USA; 5 Materials Department and Materials Research Laboratory, University of California, Santa Barbara, CA 93106, USA; 6 Department of Materials Science and Engineering, Cornell University, Ithaca, NY 14853, USA; 7 Department of Physics, Colorado School of Mines, Golden, CO 80401, USA; 8 Materials Measurement Laboratory, National Institute of Standards and Technology, Gaithersburg, MD 20899, USA; 9 Department of Physics, University of Texas at Austin, Austin, TX 78712, USA; 10 Department of Polymer Science and Engineering, University of Massachusetts, Amherst, MA 01003, USA; 11 Institute of Condensed Matter and Nanosciences, Université Catholique de Louvain, 1348 Louvain-la-Neuve, Belgium; 12 Department of Materials Science and Engineering, University of Maryland, College Park, MD 20742, USA; 13 Department of Materials Design and Innovation, University of Buffalo – The State University of New York, Buffalo, NY 14260-5030, USA; 14 Department of Materials Science and Engineering, Stanford University, Stanford, CA 94305, USA; 15 Department of Chemistry and Biochemistry, University of Maryland, College Park, MD 20742, USA; 16 Department of Metallurgical and Materials Engineering, Colorado School of Mines, Golden, CO 80401, USA; 17 Department of Materials Science and Engineering, University of Michigan, Ann Arbor, MI 48109, USA; 18 Department of Materials Science and Engineering, The Ohio State University, Columbus, OH 43210, USA

## Abstract

The Materials Genome Initiative (MGI) advanced a new paradigm for materials discovery and design, namely that the pace of new materials deployment could be accelerated through complementary efforts in theory, computation, and experiment. Along with numerous successes, new challenges are inviting researchers to refocus the efforts and approaches that were originally inspired by the MGI. In May 2017, the National Science Foundation sponsored the workshop “Advancing and Accelerating Materials Innovation Through the Synergistic Interaction among Computation, Experiment, and Theory: Opening New Frontiers” to review accomplishments that emerged from investments in science and infrastructure under the MGI, identify scientific opportunities in this new environment, examine how to effectively utilize new materials innovation infrastructure, and discuss challenges in achieving accelerated materials research through the seamless integration of experiment, computation, and theory. This article summarizes key findings from the workshop and provides perspectives that aim to guide the direction of future materials research and its translation into societal impacts.

## INTRODUCTION

In 2011, the announcement of The Materials Genome Initiative (MGI) challenged the scientific and engineering communities to accelerate the pace of materials discovery, design, and deployment by synergistically combining experiment, theory, and computation in a tightly integrated, high-throughput manner.^1^ In this approach, vast materials datasets could be generated, analyzed, and shared; researchers could collaborate across conventional boundaries to identify attributes underpinning materials functionality; and the time for the deployment of new materials could be shortened considerably. While the drive to uncover the “materials genome” is the all-encompassing goal of the MGI, the impetus to find and design new materials that solve problems and improve societal well-being has been at the heart of human advancement for thousands of years. Indeed, the materials available to us (and those that are not) affect the ways we think about, interact with, and manipulate the world around us. Prior to the Industrial Age, it was unimaginable that the coordinated movements of metals as mechanical parts, as exemplified by Charles Babbage’s difference engine or the Scheutzian calculation engine, could be used to accelerate basic computations by orders of magnitude. Similarly, the creators of such mechanical computers could not have envisioned further increases in computational power enabled by the development of semiconducting materials for transistors. Further still, those working on the Apollo 11 guidance computer would not have wagered that more than half of Earth’s population in 2018 would have devices in the palms of their hands featuring x1000 more computational power than a computer developed to guide spaceflight. Yet, progressively, materials discovery and engineering ingenuity open new frontiers for technological advancement. Today, we have realized the creation of metallic hydrogen, devised multijunction photovoltaics to exceed the Schockley-Queisser limit, succeeded in pinpoint gene editing, and developed an infrastructure that supports near instantaneous access to petabytes of information with the click of a button.

Analogous to these past developments, further pursuing design and discovery of new materials via scientific research will dictate future societal developments. Flexible biosensors could be implanted *in vivo* and harmlessly degrade when their job is done. Recyclable plastics could be created from excess carbon dioxide towards a waste-free circular materials economy. Materials that harvest static electricity and thermoelectric power derived during daily activities could be integrated to power personal electronic devices. 3D printers could print bone implants, braces, or contact lenses while visiting the doctor’s office. Advanced superconducting materials could incite development of quantum-information technologies for more advanced communication and cryptography systems. These potential developments are based on our current conception of possibilities for manipulating the physical world, which can be drastically modified by the development of new materials, much in the same way that ramifications of the Internet were not envisioned prior to the advent of the transistor.

Integral to the design of new materials will be new means of doing, recording, and sharing science. As a representative example, we envision a scenario involving the high-throughput screening of soft matter, an area of enormous promise that is not as developed as other disciplines in terms of high-throughput screening due to the inherent disorder of these materials. This scenario involves a researcher in corner A of the country submitting a query to a user facility that synthesizes and characterizes a new class of polymers in a high-throughput manner using advanced, modular robotics. The results automatically populate a centralized polymer database, reporting successful, and failed, synthetic and processing routes, alongside a set of typical materials properties. As these data are published online in a freely available, shareable and standardized data format, a computational researcher in corner B of the country uses the database of experimentally measured properties to calibrate a new computational model that predicts materials properties on the basis of molecular structure. Within an inverse-design optimization framework, that researcher submits this high-throughput computation request to a user-facility cloud computing system available on a core/hour basis to identify five chemical structures that optimize the target material property. After obtaining these results, the set of all considered molecular structures along with the five candidates, which are flagged to the community, are posted in the online database alongside the experimental results. Meanwhile, a researcher at location C with expertise in polymer processing observes both the successful and failed processing routes posted earlier and refines a data-driven model capable of predicting the optimum processing route given an input molecular structure. Having seen the flagged molecular structures from the researcher in corner B online, this last individual at location C determines three processing protocols for three of the flagged structures and places these in the database alongside the corresponding molecular structures, and the researcher at location A uses these structures to seed the next phase of their experimental search. Some elements of this vision can be addressed technically, while others require challenging traditional academic customs and incentive structures. This is one manifestation of the MGI paradigm at play in future materials research, with initial pilot programs in this vein now emerging [https://www.nist.gov/mgi].

In this article, we summarize key findings from the May 2017 workshop “Advancing and Accelerating Materials Innovation Through the Synergistic Interaction among Computation, Experiment, and Theory: Opening New Frontiers,” held at and sponsored by the National Science Foundation (NSF). The workshop brought together experts from a variety of sub-disciplines (See Appendices A and B of the Supplementary Information) to review successes from the MGI and identify future scientific opportunities for materials design and discovery. Over 100 researchers and policy-makers deliberated on the focus areas of the workshop over the year preceding the workshop. Through those discussions and based on the MGI’s ultimate goal to bring products to market, from conception to deployment, faster and more cheaply, six application-focused domains were identified via consensus as areas of importance: (i) Materials for Health and Consumer Applications, (ii) Materials for Information Technologies, (iii) New Functional Materials, (iv) Materials for Efficient Separation Processes, (v) Materials for Energy and Catalysis, and (vi) Multicomponent Materials and Additive Manufacturing. In the following, we first highlight some representative examples of MGI research and then discuss specific successes, opportunities and challenges, and aspirational perspectives as they pertain to each of the aforementioned focus areas, emphasizing facets of MGI-inspired research paradigms. Afterwards, we outline many unifying themes critical to the advancement of materials discovery, irrespective of sub-discipline. Through this conspectus, we trace the current trajectory of the MGI to new frontiers for materials discovery.

## REPRESENTATIVE EXAMPLES OF SUCCESSFUL MGI RESEARCH

**Fig. 1:**
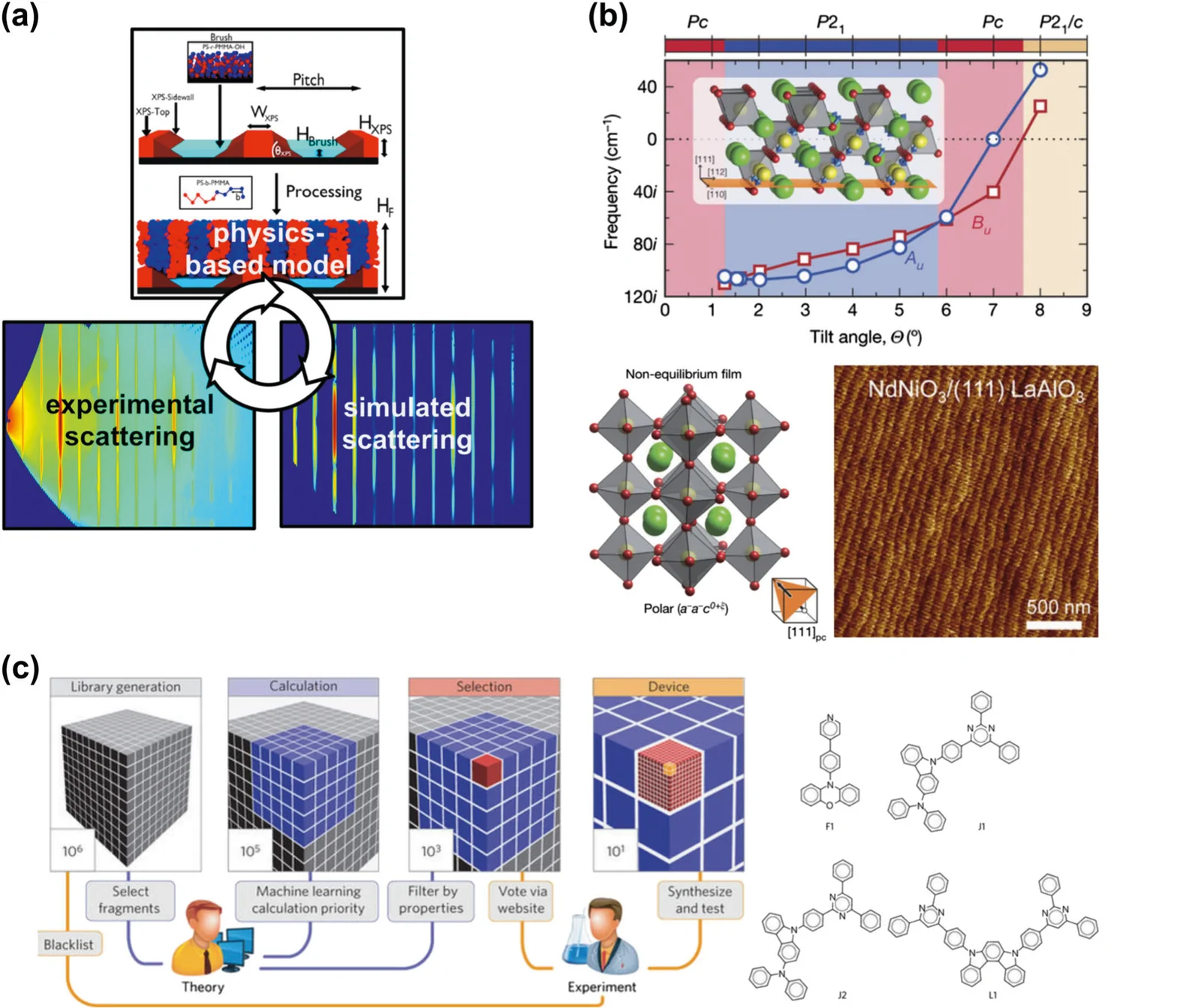
Representative examples of successful MGI research. **a** A closed-loop approach for interpreting structure-formation in block-copolymer self-assembly using physics-based modeling (Adapted with permission from ref. ^2^, Copyright American Chemical Society 2017), **b** geometric design of new polar metals using theoretical insight from ab initio simulations and high-precision experimental synthesis (Adapted with permission from ref. ^3^, Copyright Springer-Nature 2016), and **c** the design of new organic light-emitting diodes using combined high-throughput screening and experimental approaches (Adapted with permission from ref. ^4^, Copyright Springer Nature 2016)

The MGI has already enabled critical advances in materials science. Three key examples, relating to polymeric self-assembly, polar metals, and organic light-emitting diodes (OLED), are highlighted in Fig. 1. In a first example (Fig. 1a), supported by the Center for Hierarchical Materials Design, Khaira et al. combined physics-based molecular modeling, small-angle X-ray scattering, and evolutionary optimization to accurately deduce the molecular structure of experimental films in unprecedented detail.^2^ Compared to previous geometric or shape-based models, this closed-loop approach, in which physically meaningful simulation parameters are iteratively updated based on experimental feedback, which can be generated concurrently, exemplifies a new paradigm for interpreting and understanding experimental phenomena with the aid of simulation and theory. In a second example (Fig. 1b), enabled by the Designing Materials to Revolutionize and Engineer our Future (DMREF) program, Kim et al.^3^ applied quantum mechanical simulations to design, in silico, a room-temperature polar metal exhibiting unexpected stability, and then successfully synthesized this material using high-precision pulsed laser deposition. This theory-guided experimental effort revealed a new member of an exceedingly rare class of materials, which could be critical towards new technologies requiring unusual ferroelectric behavior. In a third example (Fig. 1c), Gomez-Bombarelli et al. utilized high-throughput virtual screening combining theory, quantum chemistry, machine learning, chemin-formatics and multiple methods of experimental characterization to explore a space of 1.6 million OLED molecules,^4^ resulting in a set of experimentally synthesized molecules with state-of-th-art external quantum efficiencies. This tightly integrated approach to experiment, computation, and theory is characteristic of the MGI, and represents a stepping-stone for the systematic exploration of organic chemical space in a variety of technological applications. All three examples highlight the tremendous potential of the MGI paradigm for materials discovery when experiment, computation, and theory act synergistically to design new materials with targeted properties.

The MGI paradigm has also impacted many other key application areas. Data-driven approaches have led to breakthroughs in glassy materials^5,6^ and a better characterization of the glass genome.^7^ High-throughput screening, featuring a combination of experimental and computational efforts, has been realized not only in the context of high-performance OLED materials,^4^ but also metal-organic framework (MOF)/zeolite-based porous materials,^8,9^ lithium ion conductors,^10^ photoanodes for solar fuels,^11^ and half-Heusler piezoelectric transducer materials.^12^ Data-mining has been used to identify correlations among thermoelectric material properties and home in on promising new regions of design space.^13^ All of these discoveries represent the impact of the MGI’s data-driven, integrated approach to materials discovery, and we are just beginning to observe the fruits of these investments across a variety of sub-disciplines within materials science and engineering, as is outlined throughout the remainder of this document.

## MATERIALS FOR HEALTH AND CONSUMER APPLICATIONS

Health and consumer applications feature myriad materials—including polymers, liquid crystals, complex fluids, oxide glasses, and biologically derived or biologically inspired materials—with a diverse array of functional properties that make them ubiquitous in modern life. Such materials are often referred to as “soft”. Beyond even generic advantages afforded by their wide processability, such materials are highly susceptible to the effects of external fields and, as such, can be tuned to deliver specific functions on demand. Soft materials are used at hospitals in biomedical devices and drug delivery vehicles; purchased at the supermarket as a part of foods, drugs, and personal care products; used as structural parts and protective coatings for vehicles, buildings, and infrastructure; and featured in high-end electronic devices as elements of sensors, displays, and batteries. The ability of soft materials to support mixed ion/electron conduction presents promising possibilities for soft matter bioelectronics to greatly enhance medical diagnosis and treatment technologies. Further advances related to these and other health and consumer products will be facilitated by synergistic efforts motivated by the MGI that enable successful navigation of the nearly infinite design space provided by exploitation of covalent chemistry and physical interaction, both at and far from equilibrium.

### Successes

**Fig. 2:**
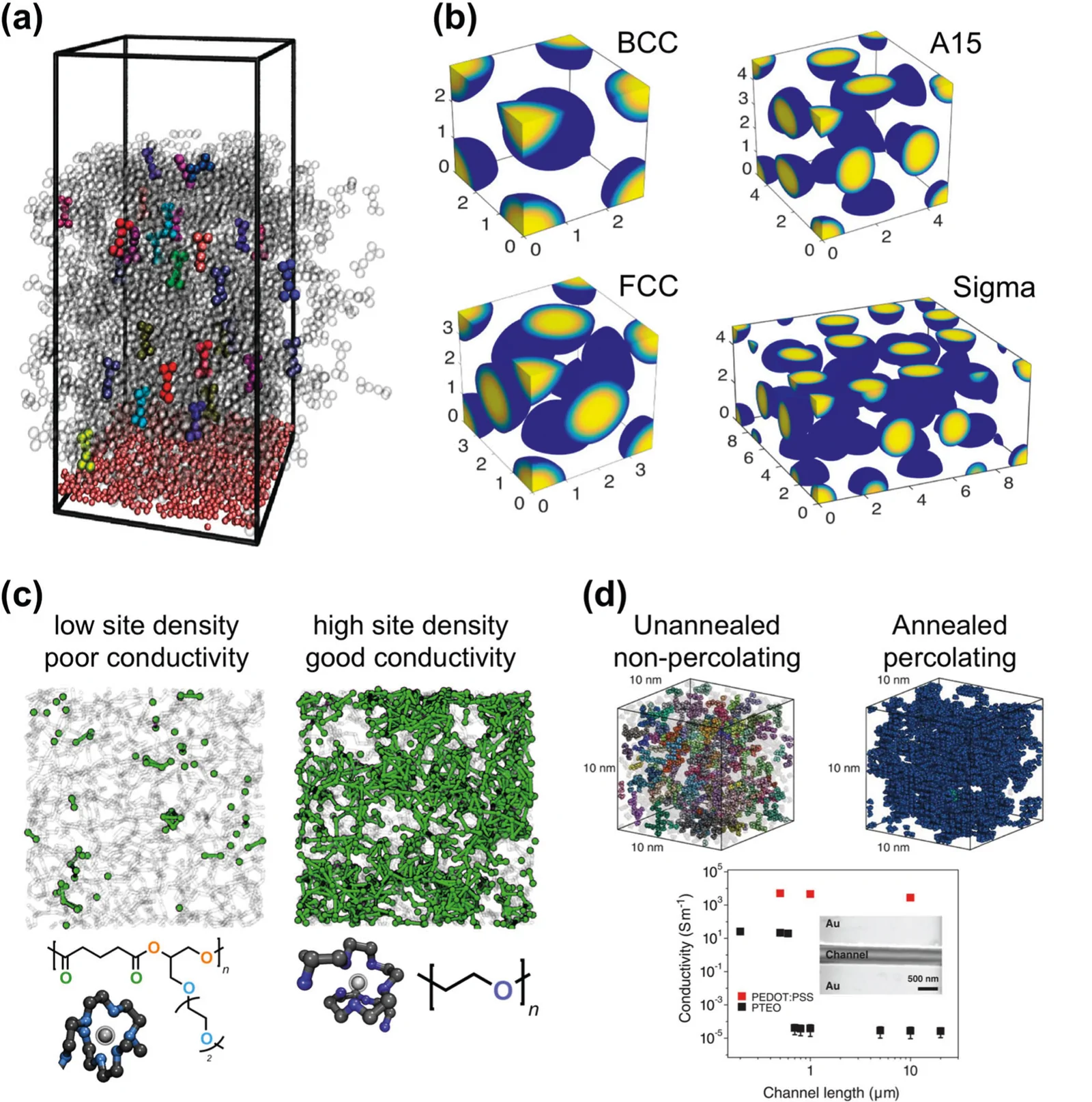
Recent accomplishments from soft matter MGI approaches. **a** Simulation of a small molecule glass highlighting oriented materials as colored (reprinted with permission from ref. ^14^, Copyright AIP Publishing 2015), **b** SCFT prediction of block copolymer phases including the Frank–Kasper *σ* and A15 sphere-forming phases (Reprinted with permission from ref. ^22^, Copyright ACS Publications 2016), **c** a comparison of simulation-derived Li^+^ solvation site networks that correlate with experimental ion conductivities (Reprinted with permission from ref. ^23^, Copyright American Chemical Society 2015), and **d** simulated structures that illustrate the percolation properties responsible for experimental electrical conductivities (Reprinted with permission from ref. 2018, Copyright AAAS 2018)

The MGI style that tightly integrates high-throughput experiment and computation has yielded critical advances in the ability to understand and tailor the physical properties of materials important to health and consumer applications. For glassy materials, which appear in a variety of industrial technologies, researchers have utilized machine learning to mine a large sampling of glass configurations to identify descriptors that strongly correlate with rearrangement dynamics^5,6^, revealing insights that did not exist prior to the MGI. Data-mining techniques applied to a vast range of systems have also led to fast and accurate models that predict bulk mechanical behavior solely from atomistic structure,^7^ and strong integration of computational modeling and experimental characterization in the MGI style has enabled the enhanced understanding of the structure and processing of organic glasses^14,15^ (Fig. 2a), leading to the development of artificially aged glasses with unprecedented stability.^16^ Moreover, there have been revolutionary advances regarding templating and fabrication of complex structures, as evidenced by the creation of complex hierarchical patterns^17,18^ and photonically active architectures^19^ using liquid crystals or the templating of two-dimensional patterns^20^ and complex lattice mesophases^21,22^ using block copolymers (Fig. 2b). Combined computational and experimental approaches have augmented our understanding of ion-transport phenomena in new polymer electrolytes^23–25^ for battery applications (Fig. 2c), further resulting in efficient screening models^26^ and new design strategies.^27,28^ Graph-based algorithms have been developed for rapidly screening the charge percolation properties of molecular networks, thus accelerating characterization of multiscale charge transport and enabling MGI data-driven screening techniques^29–32^ (Fig. 2d) The tight integration of computation and experiment has also greatly improved understanding of the behavior of charged polymer complexes, solutions, and brushes mimicking biological functionality^33–35^. These successes, along with many others, form a solid foundation for soft materials design, but many challenges still remain.

### Challenges & opportunities

#### Design theoretical and experimental techniques for characterizing non-equilibrium processes in soft materials.

Most materials for health and consumer applications are processed and used at conditions far from thermodynamic equilibrium, and this processing often imparts advantageous materials properties. Efficient simulations reporting on the formation of non-equilibrium structure combined with in situ experimental characterization techniques, which can be used to validate proposed models, would greatly accelerate the development of processing strategies and deliver a solution to the inverse design problem, as applied to soft materials. A key challenge in this area is the modeling of kinetically dependent structure formation, for which new methods are needed.

#### Extend predictive computational synthesis techniques to macromolecular synthesis.

Improving computational support for the synthesis of soft materials would be very valuable. Although significant steps have been made using machine learning to predict outcomes of simple organic reactions,^36,37^ extending this capability to include a larger range of chemical variety and macromolecular synthesis would democratize chemical synthesis and accelerate our validation of theories for new chemistries. Computational guidance in the iterative experimental synthesis process is also critical to future advances.^38^

#### Extend simulation methodologies to mimic more realistic processing conditions.

New models, methods, algorithms, and the corresponding software should be developed for simulations of soft materials at and far from equilibrium. These efforts should encompass length scales ranging from angstroms to hundreds of microns, and should strive to couple different physical processes arising across wide ranges of length and time scales. For example, capturing the behavior of defects and substrates, which are critically important to structure formation, will be necessary to provide useful guidance to experiment; it would be desirable to simulate entire heterogeneous systems of self-assembled macromolecules with complexity analogous to a cellular membrane,^39^ or even human tissue. This embodies a challenge to connect molecular descriptions of soft materials (e.g., polymer, mesogen, solvent chemistry) to mesoscale theoretical frameworks that enable efficient modeling on super-molecular lengthscales. Such connections will require advances in multiscale computations or possibly the use of materials databases that curate reliable measurements and are sufficiently populated to enable machine learning approaches for designing new chemical structures.

#### Identify the role of data for soft matter systems.

Although there is consensus that data-driven materials research will be at the forefront of future materials design, it is not clear what form this will take in the context of soft materials. While a database of excitation energies derived from electronic structure calculations may be useful in one materials context, the relevant set of calculations for designing a self-assembling biomaterial is not as simple, or the relevant order parameters or descriptors are not even known. In the context of bioelectronic materials, order of magnitude fewer materials have been investigated relative to the photovoltaic or transistor communities, and an MGI-style approach could be critical for finding new high-performance materials capable of interfacing soft matter with biomedical applications; moreover, there will be challenges associated with analyzing data produced by bioelectronics.^40^ As a community, it will be necessary to critically assess what role data can and should have when designing complex materials for health and consumer applications, identify inherent limitations imposed by the inherently disordered or inhomogeneous nature of the studied systems, and design frameworks to overcome or circumvent those limitations.

#### Understand chemically and structurally dynamic soft materials.

Engineering the dynamic rearrangement of soft matter will be crucial to create materials that respond to stimuli and provide useful functionality such as self-healing, sensing, and actuation. Some of these properties are emergent through the coupling of chemical and mechanical processes via chemomechanics and mechanophores^41,42^ or evidenced in vitrimers,^43^ but design and applications of such materials remains limited.

### Aspirational perspectives

A general soft matter inverse design solution will enable the transition of complex self-assembled materials from the bench top to the commercial marketplace. With the right tools, it should be possible not only to design materials with specific functionality, but also to down-select for materials that are amenable to scalable manufacturing methods or even including preferences for biologically derived, sustainable precursors and solvents. The result would be a rapid replacement of centuries-old incumbent materials technologies with new soft materials that offer superior functionality, lower environmental impact,^44^ and lower costs. Moreover, there are exciting possibilities for the creation of new multifunctional soft materials that will work in concert to perform complex tasks (e.g., energy harvesting, transport, sensing, and storage), or in a complementary fashion to impart multiple distinct benefits. Bioelectronics materials will integrate with and augment human biology in the form of biomedical devices, wearable sensors, or functional fabrics.^40^ Soft materials may also be harnessed for manufacturing other materials, extending the capabilities of additive manufacturing, advancing wholly new and scalable modes of hierarchical materials fabrication, reducing the energy footprint of chemical reactions, and separations by membranes.^45^ Finally, soft materials can go beyond biology by harnessing chemistries that are not possible in aqueous environments or near ambient temperatures, and by incorporating new functionalities, such as the semiconducting properties of conjugated organic photovoltaics,^46^ that exceed what is possible in nature. This may provide a framework to design soft materials that combine typically anti-correlated properties: materials that are thermally insulating but transparent, lightweight but strong, ion-conducting but rigid, or self-repairing but simple.

## COMPLEX, ARCHITECTED, AND ADAPTIVE MATERIALS

Living materials have properties and behaviors that are without analogs in currently available materials, which are traditionally passive and immutable in their functional forms. Such materials might be harnessed, repurposed, and designed to sense, respond, and interact with their environment; to heal or repair themselves after damage; to engage in autonomous and coordinated motion; or to assemble into complex, self-regulating, and adaptive multiscale architectures. These properties have tremendous potential that will underlie new technologies with impacts across a broad set of materials applications. While there have been several successes in this direction, in part paralleling and building upon forefront advances in understanding and engineering of more conventional soft materials, the design of active and adaptive materials poses new and unique challenges for realizing materials with the aforementioned functions. It is thus crucial to further understand living organisms and active matter as *materials*. Combined theoretical, computational, and experimental efforts emphasized by the MGI will be key to developing the next generation of complex, architected, and adaptive materials.

### Successes

**Fig. 3:**
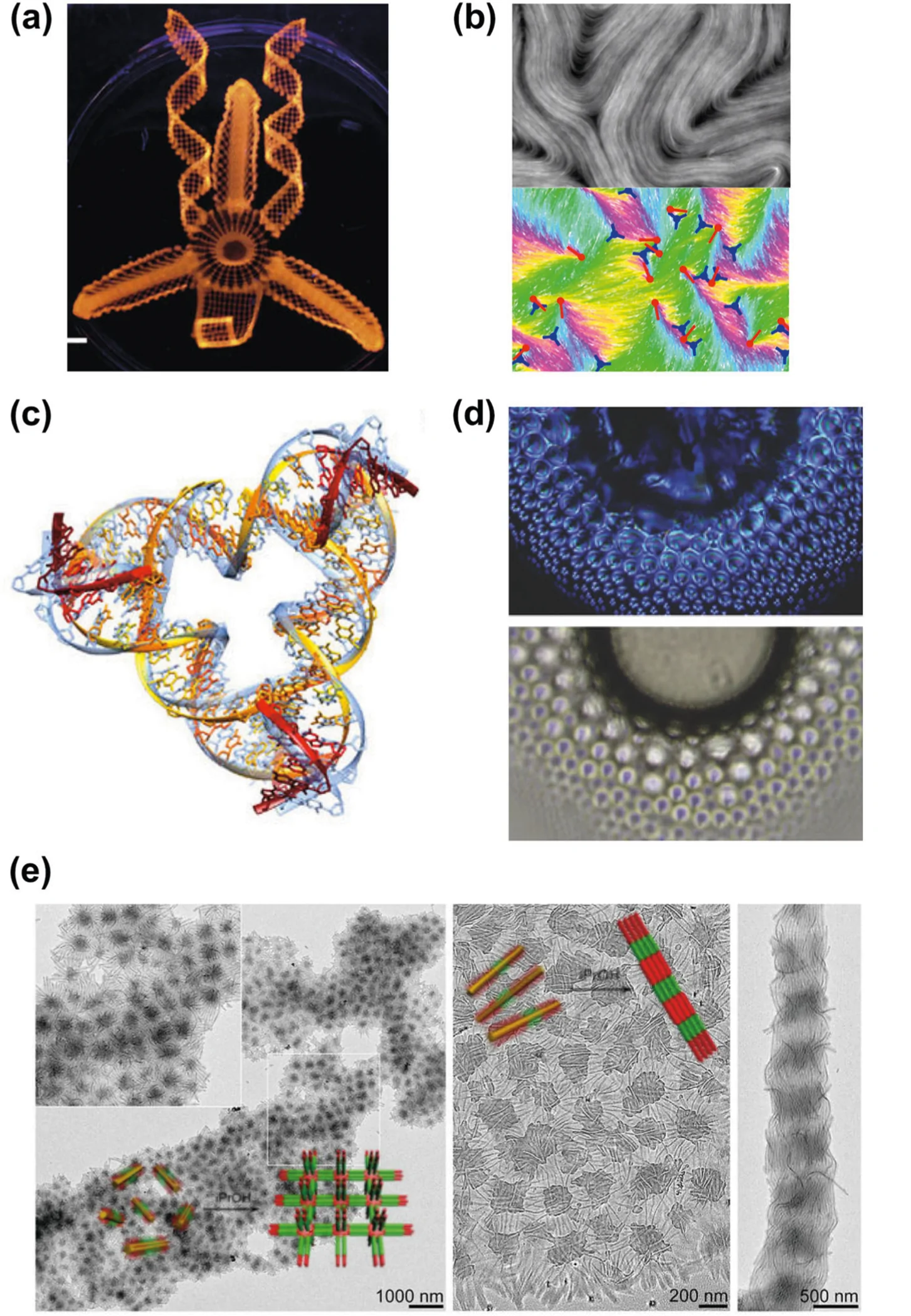
Achievements in hierarchical self-assembly of complex materials. **a** Architected soft metamaterial in the shape of an orchid (Adapted by permission from ref. ^53^, Copyright Springer Nature 2016), **b** simulated and measured spontaneous flow of topological defects in active microtubule condensates (Adapted with permission from ref. ^60^, Copyright Springer Nature 2015), **c** a 3D crystal lattice of a tensegrity motif (Reproduced from ref. ^47^), **d** self-assembly of microlenses resembling a compound eye of high sensitivity from (Reprinted by permission from ref. ^19^, Copyright John Wiley & Sons, Inc. 2015), and **e** complex 3D polymer superlattices made by harnessing crystallization, hydrophobicity, and selective solvation (Reproduced from ref. ^208^, Copyright AAAS 2015)

The MGI paradigm of tightly integrating theory, computation and experimentation has already been instrumental in advancing fundamental understanding and engineering capabilities of complex and architected soft materials to realize unprecedented materials properties. There have been incredible achievements in manipulating matter into designed structures, as exemplified by advents in DNA origami^47–49^ and DNA-modulated particulate assemblies.^50^ In addition to generating target structures, computational and inverse design frameworks advanced within the MGI have enabled the design of materials with astonishing properties, such as architected material composites with tunable negative thermal expansion^51^ or negative stiffness,^52^ mesostructured soft materials foldable into 3D shapes of nearly arbitrary complexity from purely 2D patterning^53,54^ (Fig. 3a), and atomic-scale mechanical metamaterials with previously unrealized length scale- and temperature-dependent elastic behavior.^55,56^ Liquid crystals have been designed to induce hierarchical structure in complex assemblies of colloids,^57^ knotted architectures,^58^ and templated textures.^59^ A burgeoning interest in active materials—often dense collections of self- or mutually propelling particles—has utilized the MGI’s integration of simulation and experiment to uncover emergent properties, enabling the discovery of generic mechanisms that couple hydrodynamic flows and the motion of topological defects in dense motile states^60,61^ (Fig. 3b) leading to the classification of new modes of surface instability of cohesive clusters of actively propelled particles,^62^ and the identification of coherent long range active flows in 3D active fluids.^63^ MGI design strategies have also been used to reshape the mechanical properties of soft materials through the interplay between material elasticity and geometrically engineered non-linearities/mechanical instabilities.^64–66^

### Challenges & opportunities

#### Advance “bottom-up” paradigms for materials self-assembly.

There remains a broader opportunity to augment or move beyond current fabrication techniques in order to exploit new paradigms for “programmable”^67–69^ self-assembling materials (e.g., “addressable” DNA assemblies, frustrated assembly). These approaches will lead to tailorable 3D architectures via “bottom up” processing with a level of structural and length scale control unobtainable from currently accessible “top-down” methodologies.

#### Incorporate machine learning approaches for active matter.

Complex systems driven far from equilibrium exhibit behaviors without counterpart in passive matter. Because such systems are not governed by equilibrium statistical mechanics, new methods and frameworks are needed to understand, control, and design non-equilibrium systems. With recent heightened interest in experimental active matter, there is significant opportunity to leverage the MGI paradigm and machine learning methods to analyze complex 4D data sets, extract emergent phenomena (e.g., spontaneous generation and motion of defects), and rapidly compare to phenomenological models.

#### Target autonomous & spatiotemporally regulated soft material function.

Despite recent successes in the design and engineering of 3D shape-programmable soft materials, such materials are not presently suitable for so-called soft robotics due to a lack of autonomous and spatiotemporally controlled mechanical function. Living organisms^70^ rely on the integration of two types of soft tissue functionality: neural control for signal processing and musculature for actuation motion. Advances in designing microfluidic chemical oscillator networks with the capability of regulating spatiotemporal signals^71,72^ are critical to developing actuating/responsive soft materials.

#### Encourage new innovations in conceptual theory.

The physics of topological defects in soft materials, such as liquid crystalline blue-phases and crystalline membranes, were considered the esoteric domain of theorists 30 years ago.^73,74^ However, advances in these areas are now verging to the point of engineering functional properties of soft material devices. In a similar vein, advancing the range of functional material behaviors for active matter systems will, in many cases, rely on new and yet undiscovered paradigms, which will be derived from new theoretical developments.

#### Develop materials with complexity approaching that encountered in nature.

Despite enormous advances in developing synthetic self-assembled structures, a divide remains between the complexities achieved by nature versus that achievable in the laboratory or on a computer. A biological catalyst works by structuring reactants with picometer accuracy using a complex substrate that is itself self-assembled from a protein strand; the soft materials community must extend its efforts from inert materials such as glassy block copolymers that readily self-organize, to materials that have intrinsic function, as well as order at molecular, mesoscopic, and macroscopic length scales. Advances in learning the rules of biological material composites, from ultra-tough composites like nacre to structurally colored feathers and insect bodies, parallel advances in designing and fabricating advanced and augmented materials function through 3D architectures. Nonetheless, synthetic self-assembling systems presently lag far behind biological systems in their ability to generate complex materials architectures over multiple length scales. Developing sophisticated, bottom-up self-assembly techniques, inspired by nature, will complement existing top-down approaches and facilitate the design of desirable 3D metamaterial function.

### Aspirational perspectives

Advances in designing and programming the architecture and response of soft materials hold promise for transformative technologies across materials domain. Specifically, we envision facile and scalable methods for fabricating 3D materials with programmable structure from nano to macro-scale dimensions. In contrast to the majority of existing top-down methodologies (lithography, 3D printing, or 3D weaving), these materials will be deployable via self-regulating processes that do not require environmental control. Progress made in the manufacturing of structures via 3D printing or roll-to-roll 2D processing will allow for the fabrication of customizable devices with potential applications in flexible electronics and personalized medical devices. These materials will be critical in a variety of self-sensing applications and in the reinvestment in national infrastructure via the repair and replacement of pipelines and crucial structural modalities. Lastly, developments in adaptive, architected materials will enable a new generation of deployable, autonomous and programmable soft robotics for medical interventions.

## MATERIALS FOR INFORMATION TECHNOLOGIES (IT)

Information technology is one of the greatest triumphs of 20th century science. Semiconductors enabled modern IT and are so ubiquitous in our daily lives that the present age is often called the “silicon age”. Our understanding of the physics of the semiconductor transistor began in basic research at Bell Labs, and earned Shockley, Bardeen, and Brattain the Nobel Prize in 1956. Since then, miniaturization and materials optimization have led to faster and more powerful processors, roughly keeping pace with the celebrated “Moore’s Law”. Along with this growth of processing power, parallel advances in communication and storage have enabled applications of IT to medical diagnostics, international commerce, and the construction of a communications network that connects each of us to a global community instantly.

### Successes

**Fig. 4:**
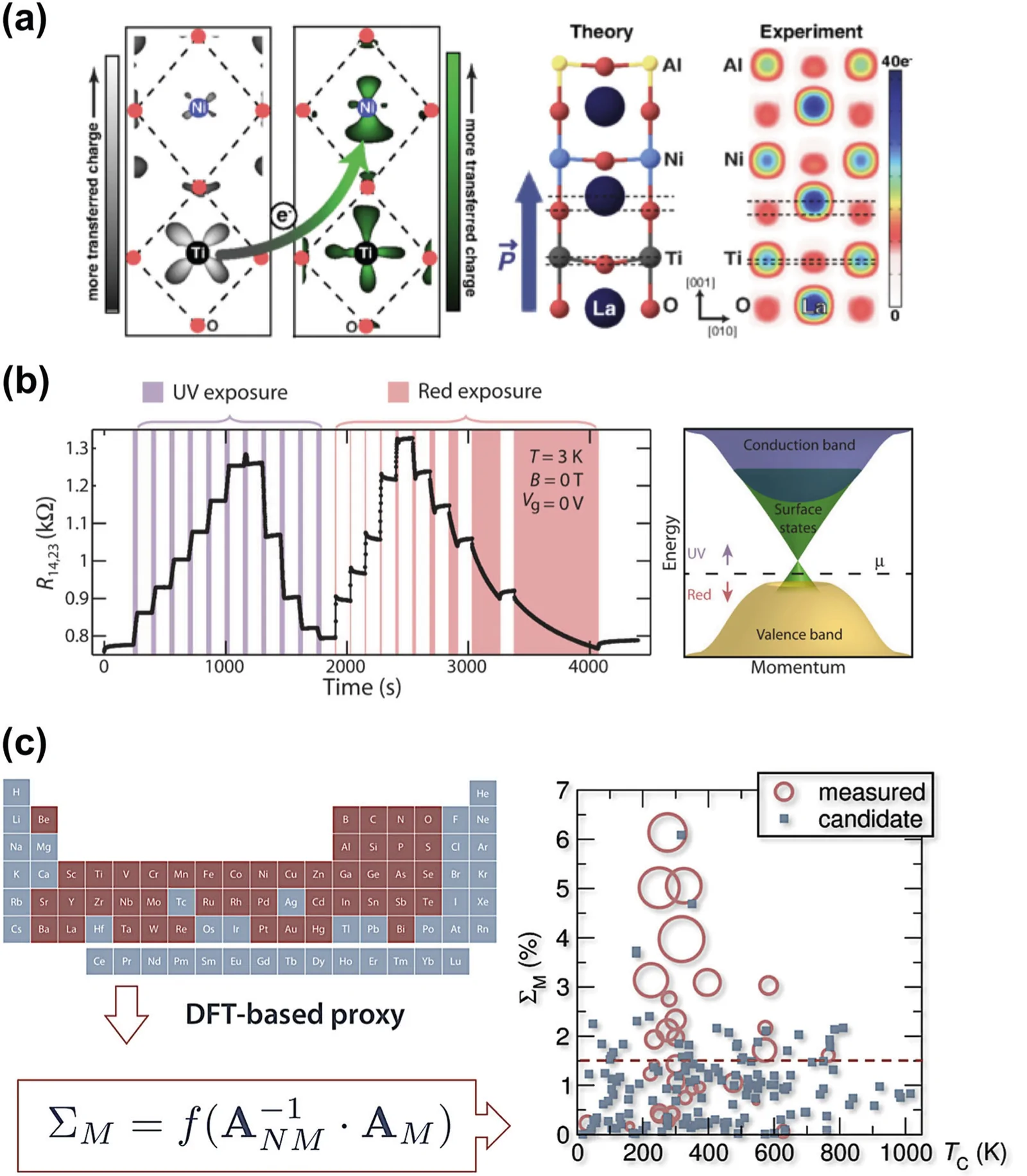
**a** DFT-calculated electron transfer and comparison of atomic structure with experimental electron density map for the orbital engineering of novel electronic systems (Reprinted with permission from ref. ^76^, Copyright American Physical Society 2015), **b** an illustration of the optical gating of a topological insulator via UV and red-light exposure (Adapted with permission from ref. ^78^), and **c** an illustration of the performance of a simple DFT-based computational proxy for screening materials based on the gravimetric entropy change upon isothermal application of a magnetic field (Adapted with permission from ref. ^80^, Copyright American Chemical Society 2017)

Both the DMREF and MRSEC (Materials Research Science and Engineering Center) programs of the NSF have had a significant impact on materials advances related to information technology. In recent work resulting from the DMREF^3^, a rare polar metal was discovered by a synergistic combination of analytical arguments, ab initio calculations, materials synthesis, and characterization constructed along the MGI paradigm of materials discovery, with the final experimental results informing further developments and refinements of the theoretical understanding of that material.^75^ In a similar vein, combined theoretical and experimental efforts enabled by the MRSEC program in the MGI style were used to predict, model, synthesize, and characterize nickel oxide systems with “buckled” lattice structures that mimic the electronic features of the copper oxides exhibiting high temperature superconductivity^76^ (Fig. 4a). Such studies motivate the search for analogs in other materials systems, which could lead to improved understanding and ultimately a room-temperature superconductor. The recent emergence of integrated studies of topological insulators has resulted in a new field of materials research and potential applications^77,78^ (Fig. 4b). Development in ferroelectric materials via the rotation of oxygen octahedral complexes,^79^ as well as the examination of magnetocaloric materials using zero-temperature magnetic deformation^80^ (Fig. 4c), have been critical in enabling potential future technologies that advance information technology infrastructure.

### Challenges and opportunities

#### Accelerate the development of new algorithms for correlated electron systems.

While ab initio density functional theory (DFT) calculation methods have advanced considerably over the past few decades, new correlated electron techniques have recently come to the fore. As one example, advances in dynamical mean field theory have become increasingly adept at capturing the influence of (especially Coulomb) interactions on a single-particle band structure. Other algorithms in this category include quantum Monte Carlo and density matrix renormalization group. The field has advanced sufficiently to predict some emergent properties of solids and could be employed more widely to study problems such as challenging metalloenzyme electronic structure and high temperature superconductivity. There is an emerging opportunity now to move these higher accuracy, full Hilbert space techniques from phenomenological models to predictions for specific materials.

#### Place greater theoretical focus on calculating response functions.

Electronic structure is critical to understanding materials but calculating response functions ties more closely to experiment and potential applications. Response functions are intrinsically more difficult because they involve correlation functions that are more sensitive to many-body effects, which are required for accurate modeling of transport and other processes crucial for IT.

#### Improve and accelerate in situ synthesis and characterization.

Recent advances, particularly in x-ray diffraction at high temperatures, pressures, and inert environments could allow a more quantitative understanding of the thermodynamics of crystal synthesis. This could improve computational models of nucleation and crystal formation, help optimize the synthesis of known materials, as well as drive the discovery of new non-equilibrium compounds that are only stable under narrow conditions. Computational models of synthesis would be helpful to encourage feedback between theory and synthesis and enable calculations of optimal synthetic conditions.

#### Leverage advances in conceptual theory with those in computational infrastructure.

Advances in theories that provide new physical insights have created new research directions, leading to the employment of computational/data/experimental driven approaches to discovering new materials. Kitaev spin liquids are a critical example, where original work began using abstract theory, followed by first-principles computational analysis, and subsequent experimental work. As advances in conceptual theory and computational methods occur, they should be integrated into emerging large-scale computational materials infrastructure.

#### Understand interface physics in correlated electron materials.

Interface physics is arguably at the core of IT materials, determining the clock-speeds, energy dissipation, and ultimate size limits on microprocessors. An emphasis on correlated electron interface physics could lead to new industrial applications of IT materials.

#### Characterize and model the impacts of disorder on materials properties.

There are not currently enough characterization tools for disorder effects nor is there sufficient investment in the theoretical understanding of disorder. Correlated materials are exceptionally sensitive to defects and imperfections, so this is a critical issue.

### Aspirational perspectives

We are rapidly approaching the physical limits of the current materials on which modern IT is built. As the size of these components decreases, quantum mechanical effects begin to dominate. One of the most detrimental effects is the increase in energy dissipation in many components, including interconnects. The next advances in IT will rely on advances in materials optimized for modern needs that are growing in both complexity and scale. The vision for the next generation of IT materials should be to enable electronics with near perfect energy efficiency and achieve highly complex computations that can model biological processes for medical applications, many-body quantum systems, traffic optimization problems, and accurate climate models. To realize this vision, new materials for IT must exploit the laws of quantum mechanics rather than be limited by them.^81^ Such a paradigm shift will be based on an exploitation of the full range of materials properties including magnetic, lattice, and orbital degrees of freedom. For example, giant magnetoresistance^82^—an effect that depends on the magnetic degrees of freedom—is widely used in computer memory (MRAM). Arguably the materials with the most promise of multi-functional behavior are those with strongly interacting electrons, which can lead to enhanced coupling between different degrees of freedom. However, these materials are the least well understood, and have a host of synthetic, characterization, and theoretical challenges.

## FUNCTIONAL MATERIALS

**Fig. 5:**
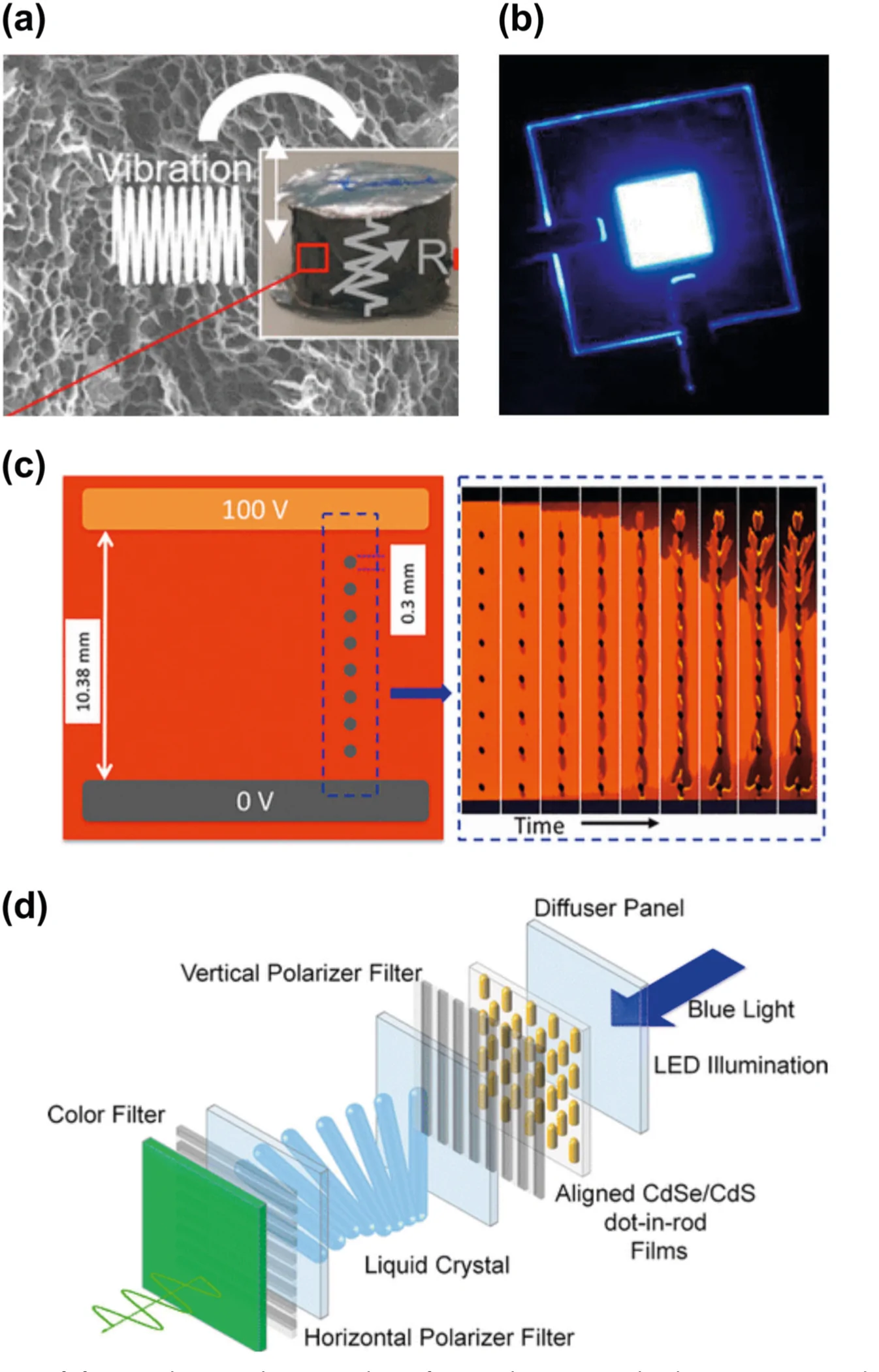
Technological impact of functional materials. A number of microelectronic and telecommunication devices involve (**a**) an accelerometer that converts mechanical constraints into an electrical signal (Adapted with permission from ref. ^209^, Copyright American Chemical Society 2017), **b** an electrochemical cell whose electron-blocking electrolyte helps convert the flow of ions into electricity (Adapted with permission from ref. ^210^, Copyright American Chemical Society 2012) **c** a liquid crystal display that modulates the polarization of light as a function of the external voltage (Adapted with permission from ref. ^211^, Copyright American Chemical Society 2017) and **d** light-emitting diodes that are coated with phosphor layers modifying the color of the emitted light (Adapted with permission from ref. ^212^, Copyright American Chemical Society 2016)

Progress in synthetic chemistry and layer-by-layer assembly has enabled the ability to design materials that respond in a prescribed way to external constraints, making it feasible to facilitate targeted functionalities by tuning their structure and composition.^83–89^ These functional materials have profoundly transformed the technological landscape, pushing the frontiers of device performance and miniaturization. Figure 5 illustrates some of the many applications, where functional materials have revolutionized our ability to communicate and navigate, to convert and/or store energy, and to process and visualize data; from piezoelectric sensing (Fig. 5a) to electrochemical energy storage (Fig. 5b) to optoelectronic displays (Figs. 5c, d).

### Successes

Under the MGI, world-class expertise has emerged in solving the inverse materials design problem to achieve a targeted functional response.^90–97^ There are numerous examples using MGI-style high-throughput techniques to explore extensive databases of compounds in the search for optimal candidates for a given technological application (Table 1), with the insights enabled by the MGI approach revealing materials properties unattainable from the use of experiment, theory, and computation independently. In addition to accelerating the discovery of functional materials, high-throughput studies provide unique opportunities to improve the predictive accuracy of existing computational models,^98^ and to refine the microscopic understanding of materials properties through the close integration of theoretical and computational research with experimentation.^99,100^ There are many successful examples of discovering new states of materials through an iterative closed-loop of experimental and computational studies,^3,101,102^ an important component of the MGI.

### Challenges and opportunities

#### Establish databases centered on interfacial and defect properties.

Materials functionalities can be dramatically influenced by interfaces and local defects. Databases established during the current MGI largely focus on bulk properties of perfect crystals; these efforts need to be expanded to interfacial and defect properties, e.g., surface energies of simple oxides and metals, ferroic domain wall energies, interfacial energies between different functional materials, organic-inorganic hybrids, and 2D heterostructures. The generation of these databases will require new instrumentation and analysis for high-throughput in operando and in situ characterization. Critical to developing interfacial and surface databases will be the use of computational methods to bridge length and timescales using data-driven approaches. Databases of computational training data can enable the generation of much faster models, e.g., establishing reaction databases from MD simulations, parameterizing classical force fields with ab initio data, or performing mesoscale phase-field simulations at larger lengthscales.

#### Shorten the time required to reproducibly synthesize and characterize new functional materials.

A key challenge in knowing whether a proposed material can be made is the lack of experimental or computational databases of reactions and kinetics for synthetic approaches. Computational reaction discovery and statistical prediction algorithms for complex processes are nascent.^103^ Moreover, there are very few existing instrumentation and analysis methods that allow high-throughput data acquisition, or in operando and in situ reproducible synthesis and characterization, for the generation of comprehensive static and dynamic property datasets.

#### Continue to increase the integration of theory, experiment, and data science.

There is a lack of systematic methods for key descriptor identification (feature selection) and machine learning from computed, measured and large-scale facility data, including systematic fashions for the reporting of model performance and baselines. Established approaches from the statistics and computer science communities combined with new methods developed specifically for materials data issues must be disseminated to the materials community, particularly as they pertain to materials science specific feature representations.^104^ Increased collaboration between disciplines will be critical for standardizing data formats relevant to functional materials, specifically computed and experimental phase diagrams.

#### Develop methods to bridge length and time scales in functional materials.

Technologically relevant properties of functional materials and devices are often determined by phenomena that occur on multiple length and time scales. For example, the responses of functional materials to external mechanical, temperature, pressure, magnetic, electric and chemical stimuli are controlled by the dynamics of the mesoscale architecture of structural, magnetic, electric polarization, charge, and chemical domains at different time and length scales. One needs to link length and time scales over orders of magnitude from atomic scale ultrafast responses to the dimension of a device and the evolution of its properties over its lifetime.

### Aspirational perspectives

Combining reliable and automated synthesis with high-throughput data acquisition and dissemination will enable identification of promising multifunctional materials capable of transforming numerous technologies. Efficient piezoelectric transducers will enable the ability to harvest excess mechanical energy from everyday activity and redirect it into portable electronics. New solid lithium electrolytes will induce order of magnitude increases in the performance of energy generation and storage technologies, allowing for electronic devices that last a week instead of a day. 2D materials could allow for the creation of new microprocessor applications capable of drastically outperforming existing CMOS (complementary metal-oxide-semiconductor) technologies, and transparent conductors will allow incorporation of these new electronics into heretofore unimagined applications. The future enabled by developments in high-performance functional materials is bright and will impact a vast array of fields common to everyday life.

## MATERIALS FOR EFFICIENT SEPARATION PROCESSES

Purification technologies pervade every aspect of modern life. Whether separating crude oil into useful constituents, purifying natural gas, or desalinating water, the scale of energy consumption involved is huge. Today, approximately 15% of the total energy consumed in the United States is used for industrial separations; this amounts to half of the total energy used by American industry.^105^ If energy inefficient separation methods, e.g., distillation, continue to be used, the energy required is expected to at least triple by 2050,^106^ i.e., about 45% of current global energy use will be needed for separation processes by 2050. It is thus critical to discover better materials for separation applications.

### Successes

**Fig. 6:**
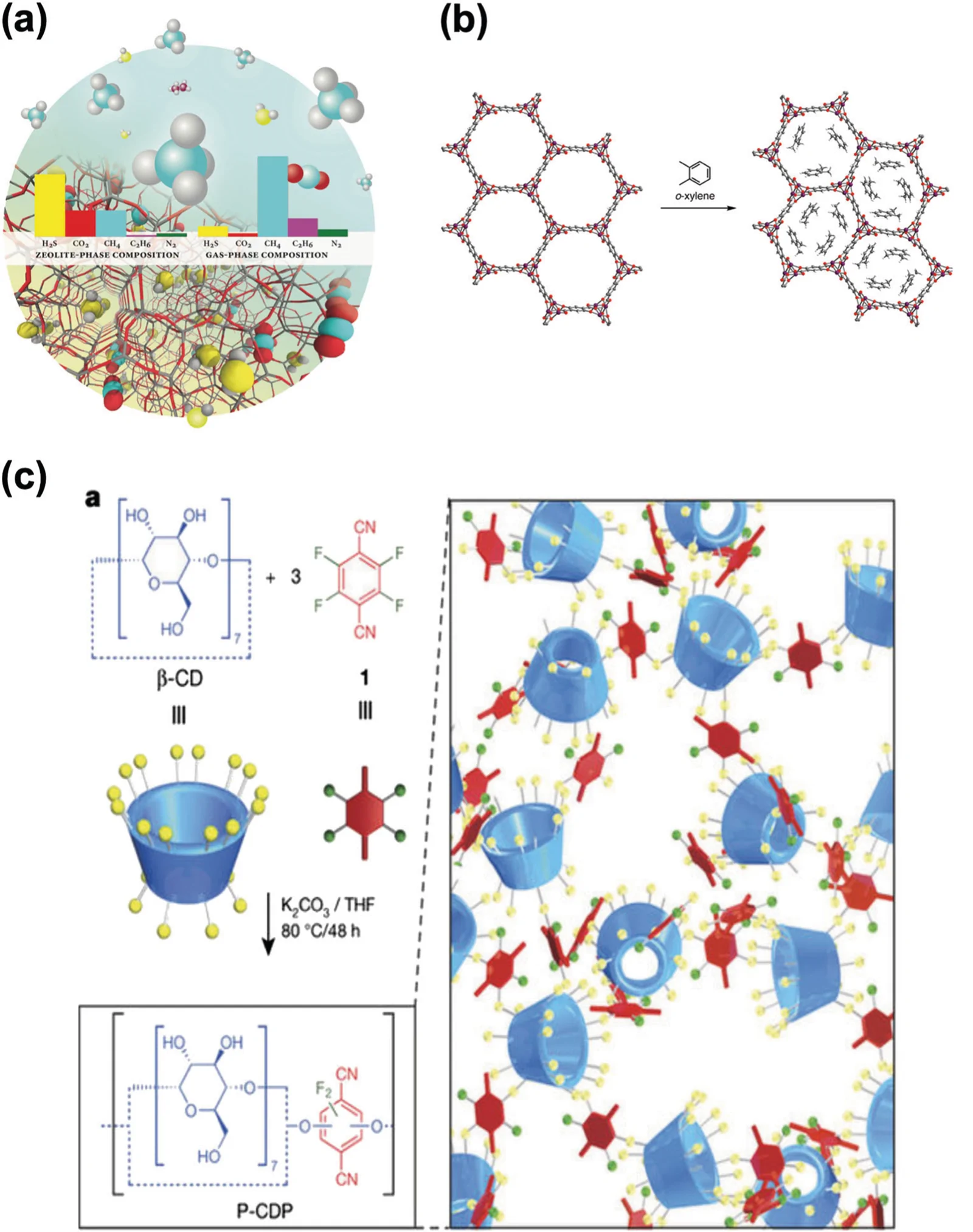
Separation materials and technologies. **a** Illustration of an adsorption-based process for the removal of H_2_S and CO_2_ from a five-component model of a sour natural gas from (Reprinted with permission from ref. ^114^, Copyright John Wiley & Sons, Inc. 2016). **b** Metal organic framework material for separation (Adapted with permission from ref. ^213^, Copyright American Chemical Society. 2018), and **c** porous polymer material for separation (Adapted with permission from ref. ^107^, Copyright Springer Nature 2016)

Harnessing modern computational and data-driven approaches in the style of the MGI has enabled several successes in the context of MOFs (metal-organic frameworks), zeolites, and other separation materials (Fig. 6). Recent work has used more than 800 previously synthesized MOF structures optimized via periodic DFT to derive critical insights pertaining to CO_2_ adsorption.^9^ Successes utilizing these computational approaches are exemplified in numerous industrial spin-offs related to separations applications. Some examples include NuMat [http://numat-tech.com/technology], which utilizes an algorithm developed by Snurr and coworkers^8^ to identify materials useful for oxygen storage, air separation, and electronic gas storage; Mosaic Materials [https://mosaicmaterials.com], which is developing low-cost synthetic methods and high-efficiency sorption processes based on highly selective sorbents developed by Jeffrey Long’s group; and Quantumscape [https://www.quantumscape.com], which aims to develop solid-state batteries aided by high-performance computation at the National Energy Research Scientific Computing Center and has secured a broad range of patents involving a variety of solid separators [Certain commercial equipment, instruments, or materials are identified in this paper to foster understanding. Such identification does not imply recommendation or endorsement by the National Institute of Standards and Technology, nor does it imply that the materials or equipment identified are necessarily the best available for the purpose.] The development of porous polymer membranes for the elimination of trace chemicals^107^ in water (Fig. 6c) and advanced adsorption technologies for the capture of pharmaceuticals in water [https://www.cyclopure.com and ref. ^108^] exemplify promising successes. These and other ventures illustrate that the MGI approach to materials discovery can succeed in industrial separations markets.

### Challenges & opportunities

#### Use theoretical approaches to resolve separations of physically similar species.

The most challenging separations target products with similar physical properties or similar molecular size and shape (e.g., O_2_ and N_2_). These separations are usually performed using molecular sieves as membrane materials,^109^ including zeolites, MOFs, and carbon molecular sieves (CMSs). These molecular sieves typically feature rigid pores that are selectively permeable to one species. The sheer size of the design space of molecular sieves, with thousands of potential candidates among zeolites, MOFs, and CMSs, invites the use of rapid computational screening combined with experiments to accelerate the process of discovery and refinement of membrane materials. While most successes for porous materials development, guided by computation and data-driven approaches, have come from focusing on sorption, challenging separation processes requiring selectivity with respect to similar species will require inclusion of the oft-ignored diffusive component to separations.^109^

#### Characterize the role of defects and interfaces in membrane materials.

For real-world applications, the sorbent or membrane materials are not a single crystal; defects, grain boundaries, and interfaces play pivotal roles in separation performance. Particularly at low loading, the presence of a few defects offering strong adsorption sites for one species can dramatically alter adsorption selectivity. Similarly, blocked pores or non-selective holes can dramatically alter membrane performance. New experiments are needed to fully characterize separation materials. Advances in simulation algorithms, force fields, and machine learning are required to connect deviations from crystallinity to changes in performance.

#### Understand separations of multicomponent mixtures.

Modern computation and data-driven approaches in chemical separations have been primarily applied to the adsorption of binary mixtures (e.g., xenon/krypton, carbon dioxide/nitrogen, and ethane/ethylene)^110–113^ in crystalline sorbents (assuming rigid structures for zeolites and MOFs). However, most separations involve additional compounds in smaller mole fractions. To date, there are few examples applying modern computation and data-driven approaches to the separation of multicomponent mixtures. It is crucial to study complex mixtures, including common contaminants, to ensure that fundamental research is relevant to industrial needs.^105^

#### Design tools to automate and predict the synthesis of membrane materials.

Membrane synthesis is a critical challenge in separations technology. In many cases, the separation material itself is not thermodynamically but kinetically stable over the set of operating conditions. A complete understanding of the driving factors for the formation of these kinetically controlled phases is lacking. It is striking that among the 300,000 theoretically proposed zeolite structures only a few hundred have been experimentally realized.^114^ Although molecular-level simulations of the entire synthetic route are not likely in the near future, ab initio simulations may shed light on elementary reaction steps. Integration of machine learning approaches with experimental databases (including failures to synthesize the desired material) may afford a path to accelerate the search for optimal protocols, as has been observed in other fields.^115^ The development of automated synthesis for generating large synthetic databases is crucial to the goal of synthetic data mining.

#### Develop robust computational methods for simulating separation.

The complexity of the molecular interactions within membranes requires accurate treatment of Van der Waals forces, which are typically absent from standard DFT approaches. Moreover, the complexity of the phase space to explore in these complex nanoporous and sometimes amorphous materials calls for the development of high performance classical force-fields^116^ as they provide computationally cheaper alternatives to DFT. The challenges in the establishment of accurate and transferable force-fields will require a move away from “manual tuning” and embrace more data-driven approaches combining machine learning and large databases of DFT (with Van der Waals interactions) computations.^117–119^ Accompanying these computational efforts, a database of experimental results is needed, especially from high-resolution structural characterization at different temperatures and pressures, to validate DFT as well as force-field results.

### Aspirational perspectives

The integration of data mining, theory, and synthesis will considerably shorten the time needed to develop new separation materials. By addressing the challenges enumerated, candidate materials for particular separations will be identified in a fraction of the time currently required, leaving researchers to pursue the most efficient syntheses and processing techniques. In academic, industrial, and national laboratories, these new advances and approaches in modeling and data analysis will help focus experimental design so that workers in those laboratories can minimize the number of costly experiments. This will be particularly impactful for the discovery of better materials in water management. Closed-loop water purification with advanced membranes could eliminate depletion of scarce water resources by water-intensive industrial processes in many parts of the US and elsewhere in the world, minimize seismic consequences from reinjection of spent fracking water, and reduce water requirements in mega cities that continue to grow in the developing world.

## MATERIALS FOR ENERGY AND CATALYSIS

A reliable supply of energy is critical to sustaining basic human needs and interactions in the modern world. Considering current practices with expected increases in population and industrialization, global energy demands are projected to increase to 26 TW (about a 40% increase from present day) by 2040 with commensurate increases in carbon dioxide emissions. With this growing concern regarding global climate change and the diminishing supplies of fossil fuels, the need to develop new energy strategies and technologies is critical. New, efficient energy materials and catalysts are bound to be at the heart of any successful transition to a clean-energy economy. In recent years, the MGI approach has enabled significant research progress in the direction of improving efficiency of solar cells, identifying catalysts for converting biomass or carbon dioxide to usable fuels and feedstocks, and optimizing the development of thermoelectric materials. Nonetheless, such efforts must be continued and intensified to make transformative impacts on both the national and global energy portfolio.

### Successes

**Fig. 7:**
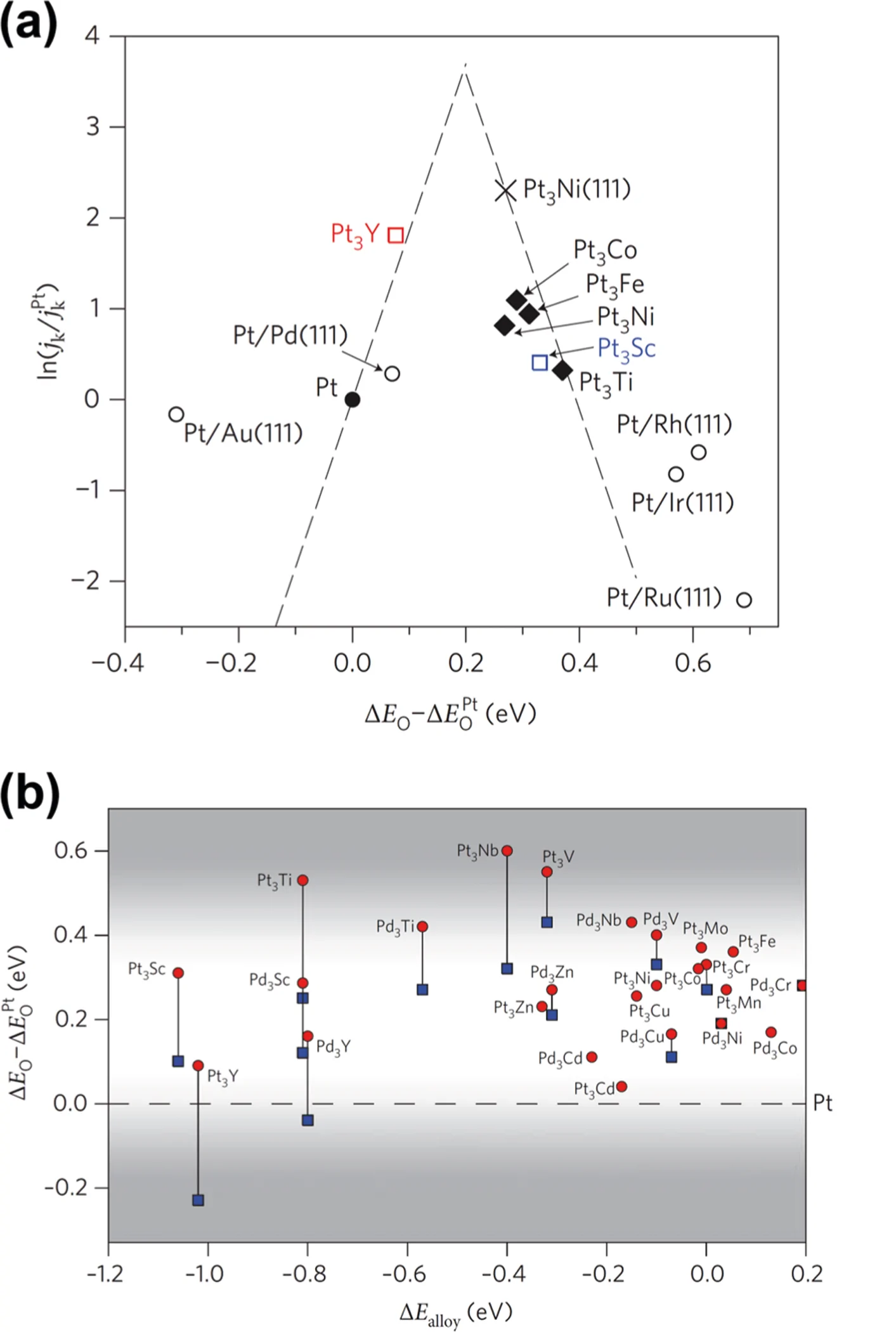
Ab-initio catalyst activity and stability screening. Results of ab initio based (**a**) activity and (**b**) stability screening for Pt-M alloy oxygen reduction catalysts (Adapted with permission from ref. ^214^, Copyright Springer Nature 2009)

Just as the fields of energy and catalysis place multifaceted demands on materials, ranging from radiation tolerance in nuclear reactors to high-capacity Li-ion battery cathodes in energy storage platforms, the impact of the MGI on energy and catalysis research has been multifaceted, promoting fundamental research and discovery alongside database construction, screening, and commercial deployment. One key reason for recent success has been due to the growing use of high-throughput ab initio methods and in silico materials design, particularly when strongly coupled to experiments and data-centered analysis as promoted through the MGI. This is embodied in efforts related to the Materials Project,^120^ which provides open web-based access to properties computed using electronic structure methods for tens of thousands of materials and chemical compounds. Use of such databases and approaches have been helpful in identifying new energy-related materials, many of which would have never been discovered without following this MGI-style approach to research.^97^ Some specific examples include (i) an oxygen reduction reaction catalyst Pt_3_Y for cost-effective fuel cells was discovered, which is 10x more active than Pt^121^ despite using three-fold less Pt for the same activity in nanoparticles^122^ (Fig. 7); (ii) at least five oxides with band structure and stability suited to provide energy efficient and stable water-splitting photocatalysts were discovered; ^123^ (iii) ultrafast ionic conductors in the Li_10 ± d_M_x_P_2-x_X_12_ family^124,125^ with liquid-like Li^+^ conductivity and low materials costs were developed; (iv) nearly a thousand highly promising organic light-emitting diode molecules were generated computationally, with some candidates achieving experimentally verified external quantum efficiencies as large as 22%^4^ and (v) multiple promising thermoelectrics from the family of half-Heusler alloys were synthesized, which can enable more efficient waste heat recovery.^126^

Many of the examples involve major contributions from industry (Samsung, Toyota, and Bosch for examples (iii)–(v), respectively), illustrating the successful application of MGI approaches in the commercial sector. In addition, combined experimental and theoretical study has greatly improved materials understanding. For example, guided by new design paradigms^127^ to break free of the limitations of existing catalysts, focused efforts to understand reaction intermediates and energetics for catalytic pathways have led to advances in water-splitting, CO_2_ reduction, nitrogen reduction, and hydrogen peroxide production.^128^ In the same vein, improved understanding of ion solvation and diffusion in battery materials has led to new design paradigms and strategies.^23–28,129^ Studies like these may constitute the foundation of future rational design efforts.

### Challenges & opportunities

#### Make materials data more discoverable, searchable, accessible, and reusable.

Developing user-friendly application programming interfaces (APIs) for databases, increasing the number and extent of databases, particularly for experimental data (e.g., full battery cycling curves at different cycling conditions or current-voltage plots for electrocatalysts, with complete experimental details embedded), and enabling searches across databases or unifying databases will greatly facilitate materials discovery. Databases can be populated by creating and/or promoting more data-oriented publications (e.g., like the Nature Publishing Group’s *Scientific Data* and Elsevier’s *Data in Brief*). Finally, machine learning can be used to generate new data missing or outside the domain of present experiments or computations. Concrete examples of these efforts are provided by Citrine [https://citrine.io/], which is aggregating data from multiple databases and making them available to integrated search and data mining, and OPTiMaDe [http://www.optimade.org/], which is making a single portal to many databases of computed properties. Both efforts support the APIs that provide convenient access for users.

#### Focus on understanding disorder, interfaces, defects, and synthetic pathways.

Most practical energetic and catalytic materials have regions of significant disorder (e.g., grain boundaries), non-trivial metastability (e.g., supersaturated dopants), hierarchical structures existing on multiple lengthscales, and dynamical processes occurring on multiple timescales. It is necessary to develop robust methods to quantify microstructure and interfaces, treat metastable non-equilibrium structures, and further develop our understanding of complex materials structure-processing-property relationships beyond bulk-phase crystals and simple interfaces. In particular, the application of MGI concepts to synthesis and processing is still in its infancy and provides only rudimentary thermodynamic guidance for those trying to make advanced materials. Moreover, the formation of defects and their influence on materials properties must be investigated to realize practical implementation.

#### Design new tools to support multiscale modeling.

New tools and understanding that can connect fundamental models to devices, including higher-dimensional defects, extreme environments, and uncertainty quantification are essential for modeling the complex coupling of relevant length and timescales. Accessible databases and automated tools that support rapid development of multiscale models from the atomic to system level are needed. The foundation of many existing studies is based on ab initio computation for small system sizes; methods to bridge the gap between these computations and realizable macroscopic properties are still needed for many applications.

#### Encourage modeling efforts to be guided by practical requirements.

Potential commercial requirements should be fully incorporated earlier in materials development research. Too often development focuses on one property, e.g., catalytic activity, without consideration of other important constraints, such as cost, toxicity, stability, etc. Slow degradation processes, e.g., deactivation of catalysts, loss of fuel cell efficiency, reduction of battery capacity, and embrittlement of nuclear materials, play critical roles in systems performance, but are very challenging to study due to the long timescales of these processes. Accelerated testing and lifetime modeling, informed by advanced modeling and data analytics, should be developed to help overcome these challenges.

#### Promote the MGI approach in other energy sectors.

Many areas of application have not yet embraced MGI approaches. For example, the petrochemical industry faces major challenges related to the science of fracking, blending fuels, methane conversion, and other areas, where MGI approaches could be transformative but are not presently employed. The increased availability of databases and machine learning tools along with improved training and education of the workforce in these areas suggests that the MGI will be valuable in these mostly unexplored application areas.

#### Develop in operando 4D characterization techniques.

The ability to dynamically observe every atom in a 3D material has long been a grand challenge of materials science. While atomistic modeling tracks every atom, limitations in accuracy and timescale have made many processes inaccessible. Experiments have reached atomic-level accuracy but have traditionally been ex situ and had many limitations, like providing two-dimensional projections or only extracting information for periodic atomic structure. With improved accuracy and scale in simulations, it may soon be possible to integrate experimental results and computational understanding to provide a complete picture of the full atomic structure of materials as a function of time.

### Aspirational perspectives

Based on MGI principles, ab initio calculations, multiscale modeling, and experimental, atomic-scale in operando 4D characterization will be combined to make transformative advances in understanding and controlling complex processes in everything from catalytic reactions to solid-electrolyte interfaces to nuclear fuel-cladding interactions. Based on mechanistic insights, new multiscale models will be developed to predict device physics under realistic operating conditions. These models and related tools can be disseminated to the broader community, and the set of predictions and conditions for various materials will be automatically scraped by centralized databases for further exploration by machine learning and data mining techniques. By addressing the aforementioned challenges, the pace at which new energy materials and catalysts are discovered—whether for batteries, photovoltaics, fuel cells, or biomass converters—will be greatly accelerated.

## MULTICOMPONENT MATERIALS AND ADDITIVE MANUFACTURING

**Fig. 8:**
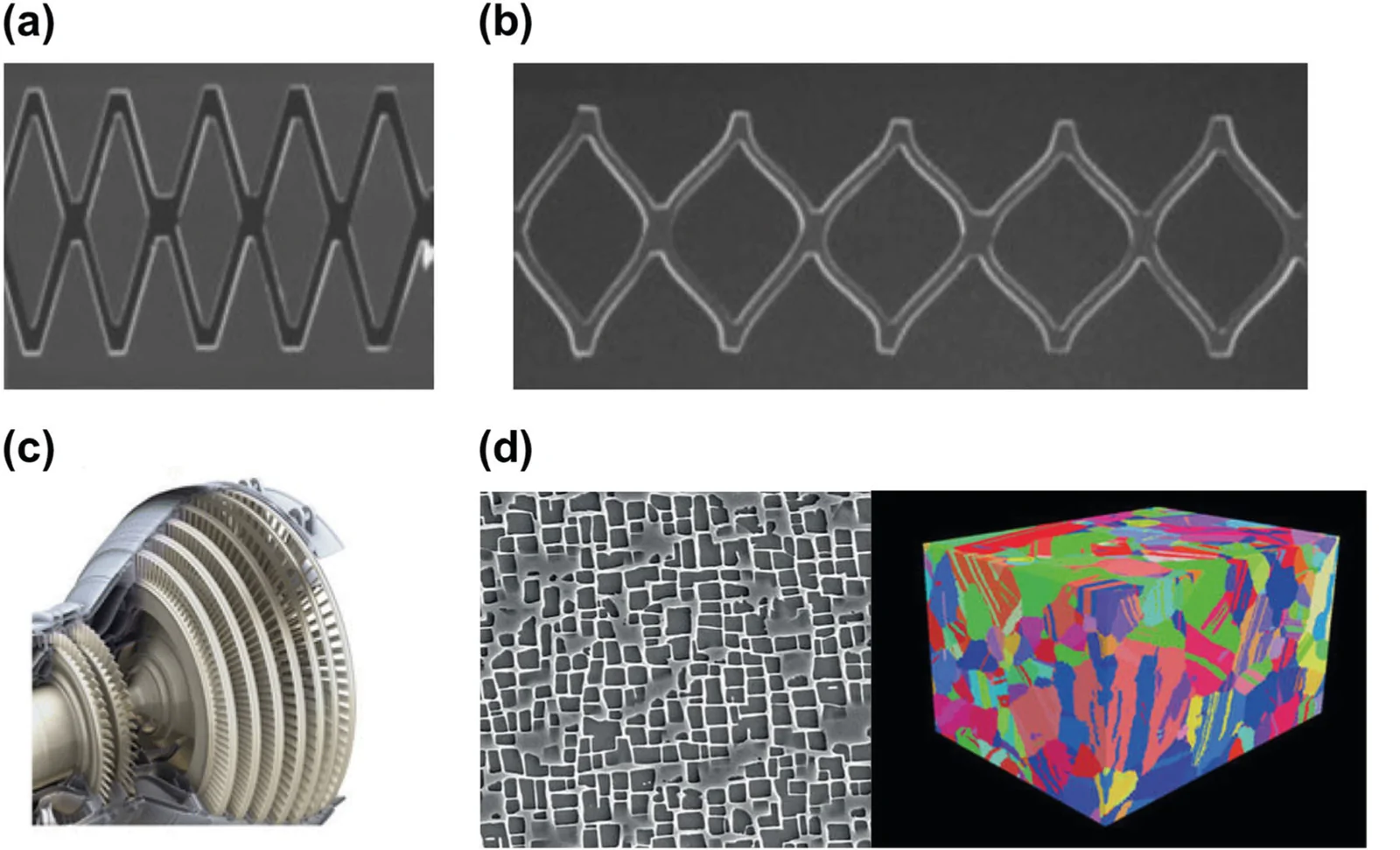
Examples of multicomponent materials and additive manufacturing. **a**, **b** A shape memory alloy with the shape depicted in (**a**) that after stretched to the shape depicted in (**b**) returns to its original shape (Reprinted with permission from ref. ^215^, Copyright John Wiley & Sons, Inc. 2018), **c** gamma TiAl turbine blade used in various Boeing models, developed using recently assembled materials databases (Adapted with permission from ref. ^133^, Copyright Springer Nature 2016), and (**d**) nickel-based materials currently in development for use in future energy-efficient, highly reliable aircraft engines: microscale view (left) and simulation (right) (Adapted with permission from ref. ^134^, Copyright Springer Nature 2016)

When considering chemistry and the potential for multiscale hierarchical structures and defects, the design space for multicomponent materials is vast. The implementation of new materials into advanced engineering systems has exhibited exciting developments (Fig. 8) but is still challenged by gaps in knowledge over these length scales. However, by drawing on the rapidly expanding menu of advanced materials and the additional promise of emerging synthetic approaches (e.g., additive manufacturing), protocols for “location specific” design may soon be within reach. This capability would be transformative for a wide spectrum of industries. Highlighted here are new materials for thermoelectrics, materials for consumer electronics, magnetic materials, battery cathode materials, fuel cell membranes, piezoelectrics, multiferroics, hydrogen storage materials, shape memory alloys, magnetocalorics, lightweight, and high temperature structural materials, and high temperature materials for aircraft engines.

### Successes

Thermodynamic databases providing the foundation for the CALPHAD (CALculation of PHAse Diagrams) approach to materials design have demonstrated the ability to accelerate the discovery, development, and introduction of new materials^130–132^ by dramatically reducing the number of experiments required for materials discovery^133^. These databases have already had a strong impact on materials in aircraft engines and other advanced aerospace vehicles,^131–134^ including a steel alloy for aircraft landing gear, several polycrystalline and single nickel alloys for power generation and aircraft engine gas turbine components.^133–136^ Databases populated by high-throughput DFT calculations have been assembled for a wide variety of multicrystalline properties, including the Materials Project (LBL/Berkeley), AFLOW (Duke), and NOMAD (EU Center of Excellence), among others.^12,90,137–140^ Additive manufacturing (AM) has impacted the 3D printing of soft materials,^141,142^ specifically for consumer health applications.^143–145^ This technology has been successfully industrialized with Kodak, Nike, and Johnson & Johnson all announcing partnerships with the Continuous Liquid Interface Production (CLIP) 3D printing company Carbon [https://www.carbon3d.com]. Additionally, powerful new tools including Dream3D,^146^ the Materials Commons data sharing platform [https://materialscommons.org], the NIST interatomic potential repository,^147^ and the Citrine Informatics machine learning platform have been developed that promise to reshape the current methods of materials discovery.

### Challenges & opportunities

#### Cultivate strategies for tailoring three-dimensional materials architectures.

The control of 3D materials architecture has the potential to generate new classes of materials. Careful tailoring of the geometrical placement of materials during synthesis can produce ultra-light weight lattices or acoustic and mechanical metamaterials that have properties not achievable via conventional bulk materials processing routes.^51,148,149^ A key enabling technology is AM, by which 3D microstructures are precisely built to induce unique properties. New platforms for 3D printing have emerged over the past decade, including powder bed processes such as Electron Beam Melting, Direct Metal Laser Sintering, and Selective Laser Sintering, and powder feed processes such as Laser Engineered Net Shaping, wire feed deposition processes, fused deposition modeling, and binder printing.^150–166^ The promise of AM is tempered by the strongly empirical approaches to tuning deposition parameters and the extraordinarily small menu of materials amenable to 3D printing. There is a dire need for predictive models that will guide the development of processing parameters and enable control of structure and defects in 3D. This is true across materials platforms, ranging from metals, to ceramics, to polymers.

#### Explore new strategies to create hierarchical 3D structures with memory.

Recent efforts have demonstrated that it is possible to design and produce through AM intricate network-based structures which retain memory of their initial state, and that can deliver highly unusual, engineered mechanical responses, including allosteric or auxetic behavior.^167,168^ Other examples have shown that it is possible to print 2D structures that subsequently fold along specific pathways to form elaborate 3D objects.^53^ Such efforts are in their infancy. A focused effort is needed, aimed at defining the range of what is possible, in terms of engineering information into a material from the moment it is created and assembled, and that encompasses computational materials design, structure engineering design, and synthesis of materials conceived to deliver specific behaviors upon printing or assembly.

#### Distribute improved techniques for data reconstruction and analysis.

**Fig. 9:**
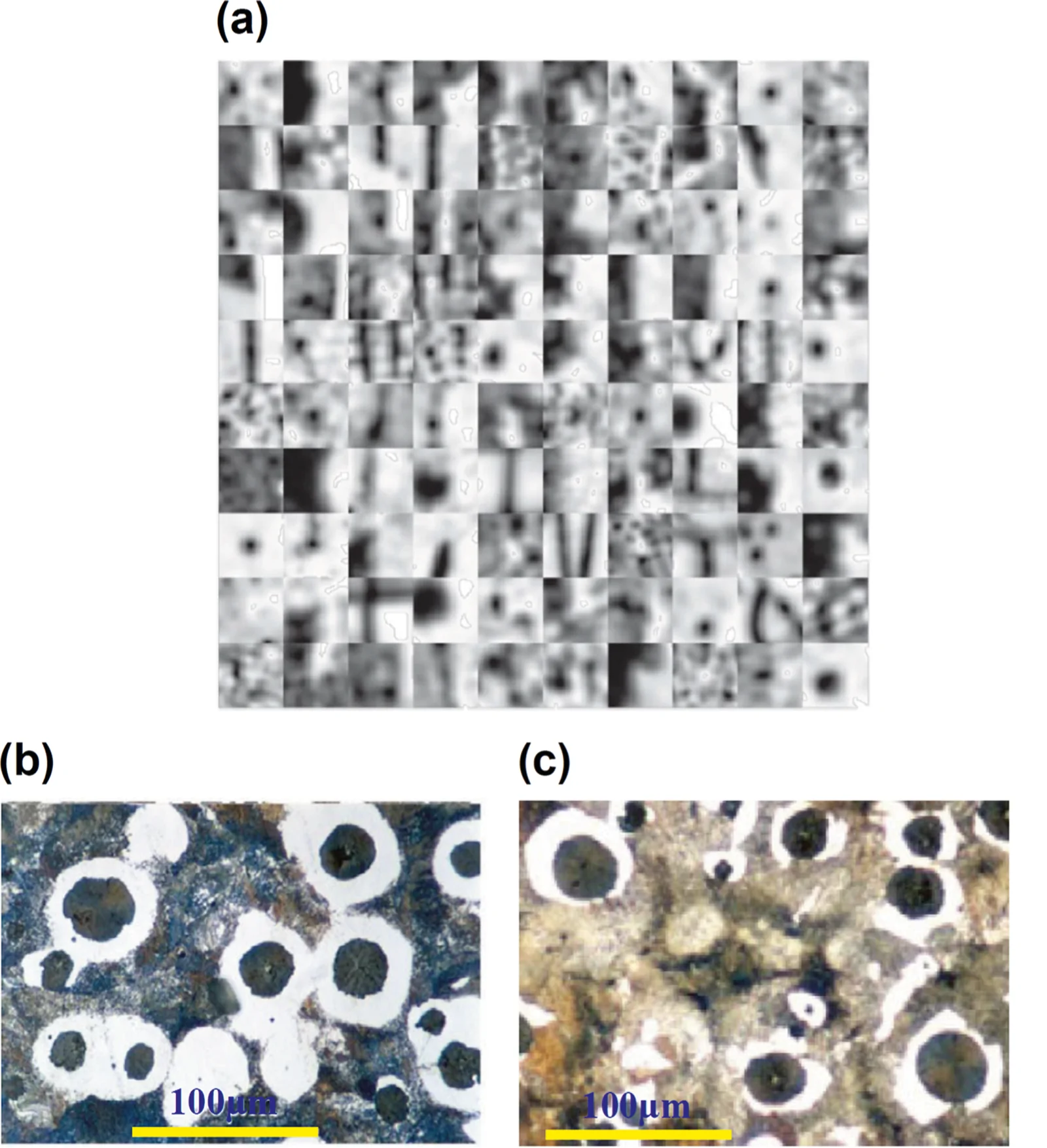
Example of computer-aided visualization and detection. (**a**) A visual dictionary used to classify microstructural data, (**b**) an example of a complex microstructural input to the computational visual classifier, and (**c**) the output of the classifier, correctly classifying the input as a ductile iron micrograph with prominent spheroidal graphite inclusions. Adapted with permission from ref. ^169^, Copyright Elsevier 2015

The rate-limiting step is no longer acquisition of materials information but data reconstruction and analysis. This data overload is apparent for AM, where layer-by-layer thermal data is now collected to track potential defect formation.^156^ An interesting early effort on the use of computer vision^169^ to autonomously classify and analyze microstructural data and identify “microstructural signals” suggests new pathways to guide development of new materials and optimize their processing, further highlighting the potential impact of advanced data tools. Terabyte-scale datasets to be captured in 3D and 4D, collected from an ever-expanding array of tomography approaches, must be effectively and efficiently collected, integrated, analyzed and shared (Fig. 9). When multimodal information is collected across different platforms, there is an added complexity of merging data from different detectors with different distortions and resolution. Significant development is needed to automate disparate materials signals for greater insight into materials structure and behavior.

#### Continue efforts for predicting behavior related to microstructure, interfaces, and the motion of dislocations.

Materials properties including strength, work hardening rate, ductility, fatigue life, fracture toughness and creep can all be drastically affected by microstructure and the presence of dislocations. Discrete dislocation dynamics (DDD) simulations are becoming more common but remain limited. While there have been insights gained in the area of small-scale plasticity and thin films,^13,90,130–135,170–174^ the challenge for the DDD simulations is to have the same impact on bulk plasticity. In the context of polymeric materials, models and characterization methods must be developed to understand welding and diffusion of polymeric molecules across interfaces, leading to entanglements and strengthening of complex three-dimensional structures as they are produced.^175^

#### Automate synthesis and characterization for multicomponent materials.

Recent approaches to use robotic arms and “slack chain” concepts to rapidly test large arrays of tensile samples are in development.^176^ With regard to fatigue, resonating samples in the kHz frequency range can reduce testing time from months to hours.^177,178^ For combinatorial/high-throughput synthesis, there have been efforts directed at fabricating materials libraries, mostly for functional materials, including coatings, catalysts, and magnetic materials,^179–182^ but also for structural materials.^183–185^ Materials discovery through combinatorial synthesis could benefit from the more standardized, low cost, high efficiency platforms and the development of “community libraries”. Optical and electronic microscopy could benefit from automated, high-throughput approaches. While high-throughput microscopy instruments are well developed for the biology community, with multiple fully automated confocal systems available,^186^ there is limited use or availability of similar instruments for the materials community. A robotic serial sectioning system^187^ has recently become available for 3D tomography, but few datasets have yet emerged.

#### Focus on the prediction of rare events in multicomponent materials.

Many properties of materials are limited by “rare features” related to microstructure or “extrinsic” defects that are either unknown or unintended. Examples include fatigue life of nickel-based turbine disk alloys, where rare combinations of grain size, orientation and the presence of annealing twins cause early strain localization and fatigue. In the context of polymeric materials for lithographic applications, defects in directed self-assembly must be reduced to levels on the order of 1 defect per 100 cm^2^.^188^ In AM, an occasional disturbance in the laser or electron beam operating conditions can result in local “lack of fusion” defects. Unintended phases, impurities or inclusions can be introduced along material processing routes for nearly every class of material. To predict materials properties at a prescribed degree of confidence, these defects must be detected and their statistical distribution quantified. While new suites of non-destructive evaluation (NDE) approaches are needed, there is a large gap between the NDE community and the materials community. Statistical approaches to predicting materials properties and their variability have also suffered from lack of cross-pollination of these communities. The methodologies for integrating multiple signals, specifically acoustic, image and X-ray signals, for detection of defects and for input to lifetime prediction models remain to be developed.

### Aspirational perspectives

The discovery of new materials with unique properties and functionalities has revolutionized entire industries (lighting, aerospace, communications, automotive), continuing the centuries-long trend for materials to fundamentally transform society (i.e., the bronze, iron and silicon ages). At this moment, materials science, long challenged by the complexity of engineering materials, is poised to be transformed by the rapid expansion of computational power, the ability to generate, archive and federate large volumes of materials information, and the emergence of informatics tools. Leveraging these developments within interdisciplinary research and development efforts will provide entirely new suites of materials that will continue the transformation of industry and society. More broadly, by fully integrating computation, experiment, and theory, critical breakthroughs may be afforded. 3D engineering objects may be manufactured on demand, with tailored and predictable properties. Materials behaviors emerging from a hierarchy of length and timescales may be predicted based on their location-specific properties, without the use of empiricism. Autonomous experiments^189,190^ and simulation platforms may generate robust new data sets for analysis. Multimodal materials signals may be collected and used for the control of synthesis, functional properties, and the assessment of materials health. Rare events in materials may be detected, predicted, and mitigated in order to guarantee materials performance.

## UNIFYING THEMES FOR MATERIALS DESIGN

While each application area or materials focus possesses unique challenges, there are needs and objectives that pervade such boundaries, resulting in overarching themes and solutions shared amongst all the previously discussed sub-disciplines. Here, we emphasize the most pressing needs that emerged during the workshop.

### Continue the integration of experiment, theory, and simulation

Despite many successful examples of the MGI approach, experiment, theory, and simulation must become even more tightly coupled to truly accelerate materials discovery. There should be continued emphasis to fully realize collaborations with true theoretical/experimental feedback, rather than separate and, at best, simultaneous experimental/theoretical study. Theoretical and computational techniques or expertise must be effectively utilized by experimentalists to guide their studies with data-driven, computation-enabled search procedures, and experimental data is needed to improve computational models. Additionally, collaborations, particularly those that encompass multiple institutions, should leverage modern cyberinfrastructure for more integrated and informed collaboration.

### Devote resources to train students in both theory and experiment

Although MGI successes have resulted in greater collaboration, there are still different jargon, working cultures, and expectations between experimentalist and computationally focused groups. While specialization will still be necessary, supplemental funding mechanisms for students to spend time in other groups or in companies to get exposure and learn the language of the experiment/theory complement would facilitate communication and raise awareness of limitations and priorities during collaboration. An environment like the NSF IGERT (Integrative Graduate Education and Research Traineeship) might foster such broad education. At the postdoctoral level, training initiatives like the NSF-funded MolSSI (Molecular Software Sciences Institute) could train students in these skills.

### Develop tools to automate synthesis and characterization

Across sub-disciplines, the need for automated synthesis and characterization techniques emerged time and again. Materials development could be accelerated greatly with the creation of high-throughput, preferably autonomous, synthetic and characterization techniques. This would provide a platform for synthetic optimization, the ability to rapidly test and inform theoretical predictions, and would work synergistically with machine learning efforts. This could be particularly impactful to construct a closed-loop that enhances synthetic discovery, using generated data to improve computational synthesis models and then using improved models to propose new syntheses. Numerous examples have yielded models for future development: medicinal industries have witnessed outstanding success in the automation of synthetic chemistry.^191–193^ Advances in flow chemistry have demonstrated potential for the automation of characterization techniques.^194^ Nanomaterials have benefited greatly from automated synthesis and characterization techniques.^195,196^ Recent work has combined deep learning for screening inorganic and metal oxide synthesis parameters.^197,198^ Future advances will likely rely on existing databases for known organic reactions,^199^ and an evolution away from the “trial-and-error” paradigm.^200^ A recent report highlights the proposed Materials Acceleration Platform (MAP) which aims to automate synthesis and characterization protocols via the use of modular robotics, machine learning, and inverse design.^201^ Companies such as the Emerald Cloud Laboratory] and Transcriptic [https://www.transcriptic.com], among a number of others,^202^ represent promising efforts towards the development of cloud-based synthesis and characterization laboratories.

### Create user facilities for high-throughput characterization

High-throughput experiments to synthesize, validate, and optimize predicted compounds have been a bottleneck for the realization of new compounds and their integration into devices and complex systems. For a single laboratory, the development of high-throughput techniques can be extremely costly and time-consuming. The need for high-throughput user facilities, such as those present at some national laboratories, is critical to the widespread generation and dissemination of data in a high-throughput manner. This mechanism requires a shift in philosophy to value the understanding of the entire materials genome for a given material in addition to perceived high-impact studies that also require facility services.

### Create opportunities for enhancing understanding of data-driven research

The amount of data in materials science is typically significantly less than those datasets for which many machine learning techniques were developed. Because datasets used by tech companies typically comprise greater than 10^5^ points, the types of systems in materials science amenable to data science are severely limited without development of high-throughput experimental and synthetic facilities. Moreover, better education and training is needed for materials scientists to appreciate the magnitude of data required to efficiently apply the techniques of data-driven research. Such training should include best practices for data acquisition, curation, and sharing, and competency in tools that facilitate them. At the graduate level and above, summer schools or programs similar to the Institute for Pure and Applied Mathematics could facilitate information exchange, collaboration, and creative problem solving. On-line courses like the NSF-supported nanoHUB,^203^ which has developed 27 courses and served approximately 75,000 learners from 1100 universities and 167 companies, should continue to be supported and disseminated. NSF-sponsored NSF Research Traineeship (NRT) programs are also enhancing workforce development by enabling programs like *Data-Enabled Discovery and Design of Energy Materials (D3EM)* at Texas A&M. Internships and exchange programs among universities, national laboratories, and industry will accelerate the information flow and the alignment of activities to increase the impact of science on society. These opportunities can range from full-time 2-year masters programs to formal online MOOCS (e.g., Georgia Tech’s Online Master of Science in Analytics) to informal Corsera / edX courses. Project-based active learning opportunities for undergraduates such as the Informatics Skunk-works at the University of Wisconsin [https://skunkworks.engr.wisc.edu], the innovative program FLAMEL at Georgia Institute of Technology [http://flamel.gatech.edu], and the new Department of Materials Design and Innovation at University of Buffalo [http://engineering.buffalo.edu/materials-design-innovation.html] are all recent initiatives that largely owe their existence to the MGI. We should extend such training efforts to include those outside traditional four-year colleges and graduate schools, e.g., community college students and high-school students, to support either their eventual enrollment in more traditional materials programs or their placement in high-quality jobs.

### Distribute tools for automating, collecting, curating, and sharing data

A concerted effort must be made to design tools for materials scientists that automate the collection, curation, and distribution of datasets. Similarly, tools for automating data extraction from the literature are critical.^204^ Some exemplary efforts in this direction include the CHIMAD Polymer Property Predictor Database [http://pppdb.uchicago.edu] and UW-Madison’s Materials Simulation Toolkit for Machine Learning (MAST-ML) [https://pythonhosted.org/MAST]. Tools should be capable of interfacing with existing databases and incorporate tutorials and workshops to raise awareness of the capabilities of these methods and train users in best practices.

### Standardize data and metadata formats

There are no standardized formats for reporting experimental or computational data in the vast majority of journals. This inhibits data scraping and text mining tools from populating databases using existing literature data. Mandating formats for data and metadata^205^ would facilitate data aggregation from the literature, thereby permitting application of data-driven approaches, as well as enhance reproducibility of experiments and simulations. The responsibility to provide metadata is currently on the individual and typically ignored; pressure provided by funding agencies, journals, reviewers, and database developers should be applied, where appropriate, to institute necessary standards.

### Incentivize and encourage the publication and sharing of null results

Most journals, and by extension most researchers, are primarily concerned with positive results. However, null results are critical to understanding material properties,^115^ yet they are infrequently disseminated. For both materials and synthetic optimization, null results define part of the materials genome, and significant resources might be used to identify synthetic conditions or compositions that are already known (by few) to be poor. There should be efforts and outlets to publish such null results, thus making so-called ‘’dark data” public. Data-oriented publications are beginning to address this issue, but incentives are needed for the academic community to obtain and share data between groups, ideally in a manner corresponding to the FAIR (Findable, Accessible, Interoperable, and Reusable) data guidelines.^206^ A recent MGI report provides a promising outline for future directions in data sharing practices.^207^

### Broaden database focus beyond bulk material properties

While databases regarding common bulk properties are essential, there are a number of other properties that are also crucial for materials design. Among these are interfacial and surface properties, which are inadequately characterized and poorly understood. Additionally, databases concerning functional properties are vital to the discovery of new materials, but equally important are databases of those material properties essential to industrial adaptations, which are often ignored in many research efforts, such as materials cost, toxicity, solubility, and stability.

### Increase interaction with industrial partners

There should be support for direct interaction between Ph.D. students and industrial partners. Industry representatives at the MGI workshop identified three key traits that are highly desirable in prospective employees: (1) can the candidate work independently and do they have the important technical skills? (2) Can the candidate work effectively across theoretical, experimental, and data-centric boundaries? (3) Can the candidate adapt rapidly to new techniques required by a fast-changing research landscape? We should introduce realistic industrial needs into integrated CET (certified engineering technologist) training through encouraging collaboration with companies, using industry relevant examples, including system relevant considerations outside of basic material properties, and engaging with documents outside the academic literature, e.g., patents. Connections to the interest of industry can be improved. Certain sectors of US industry have historically had a strong presence in fundamental research; notable examples include Bell labs, IBM, Kodak, Google Accelerated Sciences, and Xerox. The needs of these industry sectors are not well known to the academic community, pushing industry to look beyond the US for expertise. It is thus of broad economic importance to make better connections to industry, including the participation of industry scientists in academic conferences and workshops, as well as longer term collaborations involving student exchanges, internships, and multi-PI grant structures that include industry.

## CONCLUSIONS

The initial phase of the MGI widely impacted scientific and engineering research in a variety of vital industrial sectors, including medicine, energy, catalysis, transportation, and computing, and it is now time to explore new frontiers for materials design. The challenges and opportunities outlined herein describe a range of critical material needs that can be addressed by further investment in the MGI principles. Through the continuing application of data-driven, integrated efforts in experiment, theory, and computation, the underlying structure-function relationships intrinsic to the materials genome can be revealed. By applying these insights to generate extraordinary advances capable of sparking revolutionary new technologies, the Materials Genome Initiative will seed a new generation of advanced materials and materials innovators capable of transformative impact.

## Supplementary Material

Supplementary information

**Supplementary Information** accompanies the paper on the *npj Computational Materials* website (https://doi.org/10.1038/s41524-019-0173-4).

## Figures and Tables

**Table 1.** Examples of high-throughput MGI-style approaches to materials design

Search target	Search space	Count	Search criteria	Search result	Ref.
Piezoelectric transducers	Ternaries with half-Heusler structure	~1000	Local and relative stability, Band gap, Piezoelectric constant, Electromechanical coupling	~80 compounds, including KMgP, LiNaS, MgBaSi, MgBeGe, NaZnP, NaZnAs, KMgSb, NaKO, KMgAs, LiNaSe, AgSrAs, CuSrAs	^13^
Piezoelectric transducers	BaTi0_3_-based solid solutions	~1200	Verticality of phase boundary (rhombohedral–tetragonal)^◊^	(Ba_0.5_Ca_0.5_)TiO_3_–Ba(Ti_0.7_Zr_0.3_)O_3_^II^	^110^
Shape memory alloys	Ti_0.5_Ni_0.5*–x–y–z*_Cu*_x_*Fe*_y_*Pd*_z_* alloys	~10^6^	Width of thermal hysteresis (experimentally trained model using theoretical structural, chemical, bonding features)	~35 compounds, including Ti_50_Ni_46.7_Cu_0_._8_Fe_2.3_pd_0.2_^II^, Ti_50_Ni_48.1_Cu_0.2_Fe_1.5_Pd_0.2_^II^, Ti_50_Ni_46.5_Cu_1.1_Fe_2.2_Pd_0.2_^II^	^111^
Electroactive molecules	Quinone derivatives with 1–3 rings	~1700	Energy of formation and solvation, Redox potential	~300 molecules	^112^
Fluorescent molecules	Donor–bridge–acceptor organics (D–B–A)	~10^6^	Vertical absorption energy (S_1_), Singlet–triplet gap (S_1_–T_1_), Oscillator strength (S_1_–S_0_)	~1000 molecules, including 4 molecules^II^ based on carbazole/ phenozazine (D), benzene (B), pyrimidine/ pyridine (A)	^5^
Photocatalytic electrodes	V0_4_-based ternaries	~170	Energy of formation, Band gap, Band edge, UV-vis threshold^I^, Photocurrent^I^	~15 compounds, including Ag_3_VO_4_^II^, CrVO_4_^II^, CoV_2_O_6_^II^, Cr_2_V_4_O_13_ (2 variants)^II^	^12^
Photovoltaic absorbers	Generalized chalcopyrites	~260	Spectroscopic limited maximum efficiency (aggregate of band-gap and absorption-spectrum features)	~20 compounds, including AgIn_5_Se_8_, Cs_3_AlTe_3_, Cu_3_TlS_2_, Cu_3_TlSe_2_, Cu_7_TlS_4_ (3 variants)	^113^
Transparent conductors	Binary, ternary, and quaternary oxides	~3000	Band gap, Effective mass, Dopability	~20 compounds, including K_2_Sn_2_O_3_, B_6_O, Ba_2_BiTaO_6_.	^114^
Transparent conductors	Ternaries with half-Heusler structure	~480	Energy of decomposition, Band gap	TiCoSb, TiRhSb, TiIrSb, ZrRhSb, ZrIrSb, HfCoSb, HfPtSn, TaIrGe^⊙^, TaIrSn	^115^
Solid Li electrolytes	Li-containing inorganic crystals	12,831	Ionic conductivity, electrical conductivity, oxidation potential, structural stability, material cost,	21 inorganic crystals	^116^
Thermoelectrics	Half-Heusler Alloys	28	Electrifical conductivity, Seebeck coefficient, electronic thermal conductivity	Nb-Co-Sn	^117^

I: Determined experimentally

II: Synthesized and confirmed experimentally

Table containing examples of successful MGI style materials discovery in the areas of piezoelectric transducers, shape memory alloys, electroactive molecules, fluorescent molecules, photocatalytic electrodes, photovoltaic absorbers, transparent conductors, solid Li electrolytes, and thermoelectrics
