# Supplementary material for: New frontiers for the materials genome initiative
Source: NPJ Comput Mater. Author manuscript; Available in PMC 2026 Sep 16. (PMC13576042; doi:10.1038/s41524-019-0173-4)
Supplement: Supplementary file 1 [file NIHMS1588384-supplement-Supplementary_information.docx]

**Supplementary Information For**

**New Frontiers for the Materials Genome Initiative**

Juan J. de Pablo^1,+^, Nicholas E. Jackson^1^, Michael A. Webb^1^, Long-Qing Chen^2^, Joel E. Moore^3^, Dane Morgan^4^, Ryan Jacobs^4^, Tresa Pollock^5^, Darrell Schlom^6^, Eric S. Toberer^7^, James Analytis^3^, Ismaila Dabo^2^ , Dean M. DeLongchamp^8^, Gregory A. Fiete^9^, Gregory M. Grason^10^, Geoffroy Hautier^11^, Yifei Mo^12^, Krishna Rajan^13^, Evan J. Reed^14^, Efrain Rodriguez^15^, Vladan Stevanovic^16^, Jin Suntivich^6^, Katsuyo Thornton^17^, Ji-Cheng Zhao^18^

**APPENDIX A. NAMES OF PLENARY SPEAKERS AND THEIR AFFILIATIONS**

**Materials for Health and Consumer Applications**. Randall Kamien (University of Pennsylvania), John Mauro (Corning), Christopher Spadaccini (LLNL)

**Materials for Information Technologies.** Charles Ahn (Yale University)

**Functional Materials**. Alan Aspuru-Guzik (Harvard University)

**New Materials to Enable Energy Efficient Separation Processes**. William Koros (Georgia Tech)

**Energy and Catalysis**. Jeffrey Greeley (Purdue University)

**Multicomponent Materials and Additive Manufacturing**. Elizabeth Holm (Carnegie Mellon University), Peter Voorhees (Northwestern University)

**Cyberinfrastructure and Data**. Bryce Meredig (Citrine Informatics)

**APPENDIX B. NAMES OF PARTICIPANTS AND THEIR AFFILIATIONS**

**Materials for Health and Consumer Applications.** *Dean DeLongchamp* (NIST), Greg *Grason* (UMass – Amherst), Andrea Liu (University of Pennsylvania), Juan de Pablo (University of Chicago), Nick Jackson (University of Chicago), Michael Webb (University of Chicago), Lisa Hall (Ohio State University), Anna Balazs (University of Pittsburgh), Venkat Ganesan (University of Texas – Austin), Andrey Dobrynin (University of Akron), Mark Ediger (University of Wisconsin – Madison), Eric Furst (University of Delaware), Kathryn Beers (NIST), Patrick Mather (Bucknell University), Ivan Smalyukh (University of Colorado), Emmanuela Del Gado (Georgetown University)

**Materials for Information Technologies**. *Greg Fiete* (University of Texas), *James Analytis* (UC Berkeley), Joel Moore (UC Berkeley), Kristin Persson (LBL), Craig Fennie (Cornell University), Ichiro Takeuchi (University of Maryland), Stephen Wilson (UC Santa Barbara), Jed Pitera (IBM), Elif Ertekin (University of Illinois)

**Functional Materials**. *Ismaila Dabo* (Penn State University), *Evan Reed* (Stanford University), Long-Qing Chen (Penn State University), Sergei Kalinin (ORNL), Kasra Momeni (Lousiana Tech), Jiamian Hu (University of Wisconsin – Madison), Turab Lookman (LANL), Karin Rabe (Rutgers University), Jeffrey Neaton (LBL), Nicole Benedek (Cornell University)

**New Materials to Enable Energy Efficient Separation Processes**. *Geoffroy Hautier* (Université Catholique de Louvain), *Efrain Rodriguez* (University of Maryland), Darrell Schlom (Cornell University) J. Ilja Siepmann (University of Minnesota), Daeyeon Lee (University of Pennsylvania), Andrew Ferguson (University of Illinois), Julia Mundy (UC Berkeley), Paul Forster (UNLV), Venkat Vishwanath (Carnegie Mellon University), Eric Lin (NIST), Christopher Soles (NIST), Robert Bedard (Honeywell)

**Energy and Catalysis**. *Jin Suntivich (*Cornell University), *Yifei Mo* (University of Maryland), Dane Morgan (University of Wisconsin – Madison), Ryan Jacobs (University of Wisconsin – Madison), Yue Qi (Michigan State University), Shyue Ping Ong (UC San Diego), Yelena Gorlin (Bosch LLC), Kristen Fichthorn (Penn State University), Boris Kozinsky (Bosch LLC), Maria Chan (Argonne National Laboratory), Daniel Cogswell (ARAMCO), Ryan Dehoff (ORNL), Kirk Gerdes (NETL), Brett Savoie (Purdue University)

**Multicomponent Materials and Additive Manufacturing.** *Katsuyo Thornton* (University of Michigan), *Ji-Cheng Zhao* (Ohio State University), Tresa Pollock (UC Santa Barbara), Michael Titus (Purdue University), Amy Clarke (Colorado Schools of Mines), Blas Pedro Uberuaga (LANL), Samantha Daly (UC Santa Barbara), Veera Sundararaghavan (University of Michigan), James Warren (NIST), Carelyn Campbell (NIST), Will Lenthe (UC Santa Barbara)

**Cyberinfrastructure and Data**. *Krishna Rajan* (University of Buffalo), *Vladan Stevanovic* (Colorado Schools of Mines), Eric Toberer (Colorado School of Mines), Zachary Trautt (NIST), Michael Toney (SLAC), David Johnson (University of Oregon), Jason Hattrick-Simpers (NIST), Ankit Agrawal (Northwestern University), Hakizumwami Birali Runesha (University of Chicago), Ian Foster (University of Chicago), Taylor Sparks (University of Utah), Caleb Philips (NREL), Alejandro Strachan (Purdue University).

**Subsection coordinators are shown in italics*.
